# Supplementary figures and images for: Cell death and iron deposition in the liver in two murine models of acute radiation syndrome
Source: PLoS One. 2025 May 29;20(5):e0324361. doi: 10.1371/journal.pone.0324361 (PMC12121821; doi:10.1371/journal.pone.0324361)

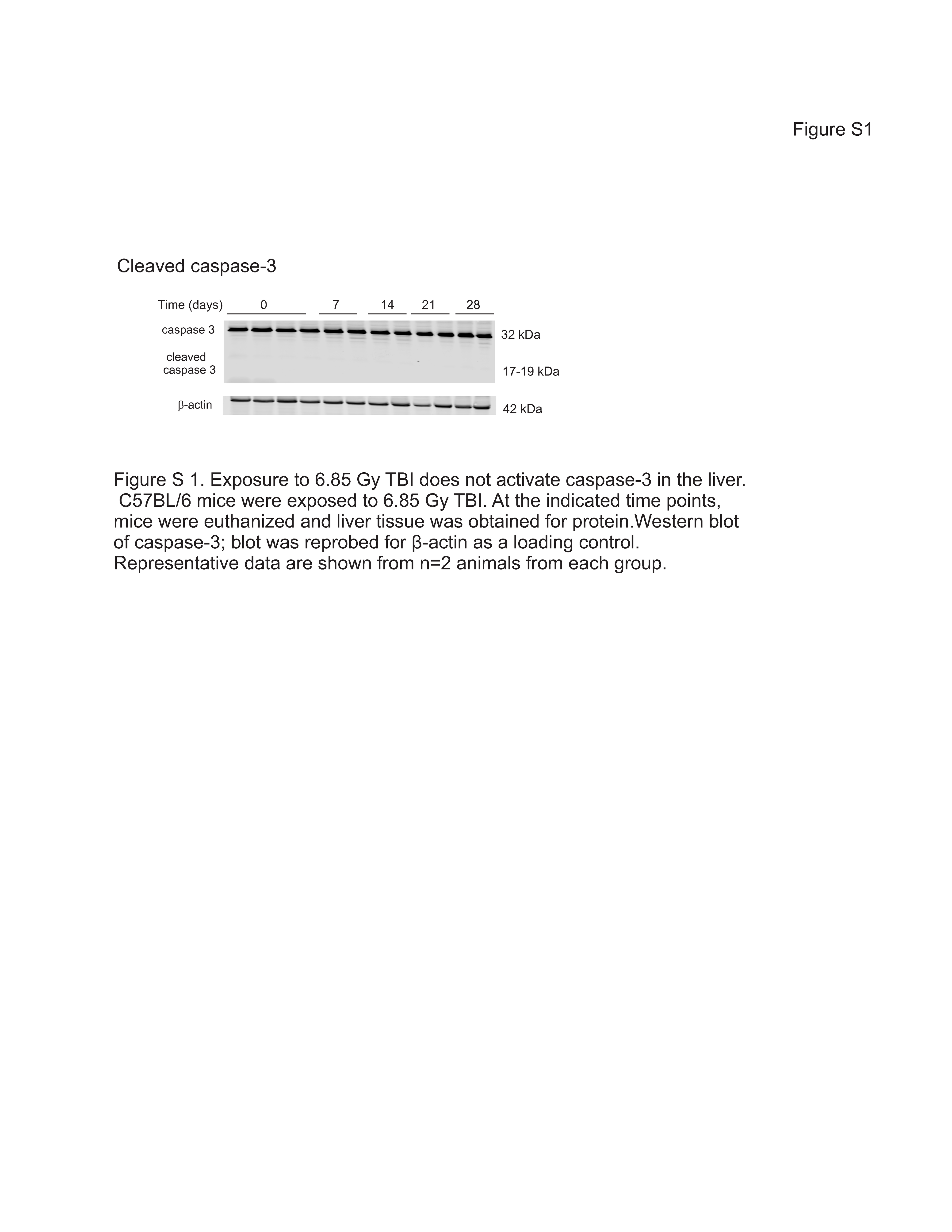

Supplement: S1 Fig — C57BL/6 mice were exposed to 6.85 Gy TBI. At the indicated time points, mice were euthanized and liver tissue was obtained for protein analysis. Western blots were performed for total and activated caspase-3; blots were reprobed for β-actin as a loading control. Representative data are shown from n = 4 control (sham-irradiated) animals and n = 2 animals from each time point post-irradiation. (TIF) [file pone.0324361.s001.tif]
