# Supplementary material for: Cell death and iron deposition in the liver in two murine models of acute radiation syndrome
Source: PLoS One. 2025 May 29;20(5):e0324361. doi: 10.1371/journal.pone.0324361 (PMC12121821; doi:10.1371/journal.pone.0324361)
Supplement: S2 Raw Images — (PDF) [file pone.0324361.s002.pdf]

7.85-7.9 Gy

Ferritin gels

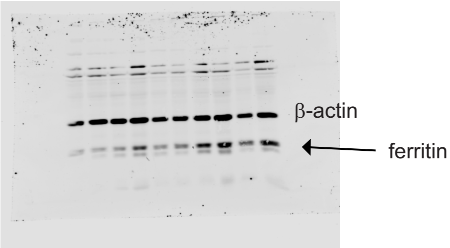

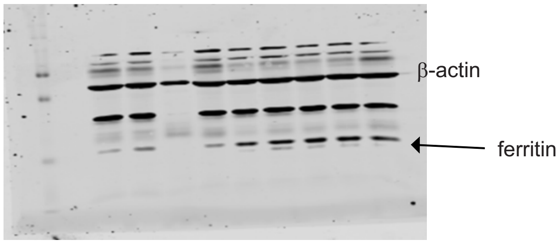

Ferroportin gels

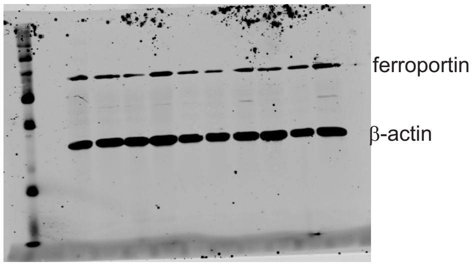

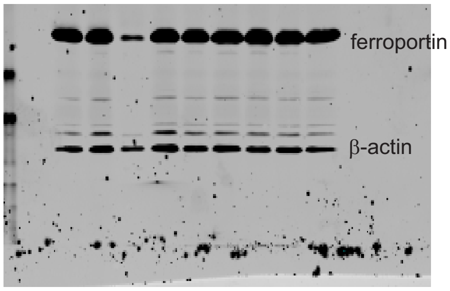

GPX-4 gels

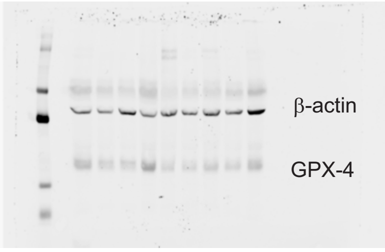

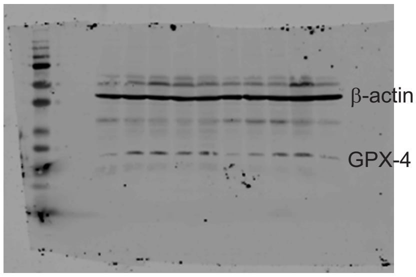

Cleaved caspase-3 gels

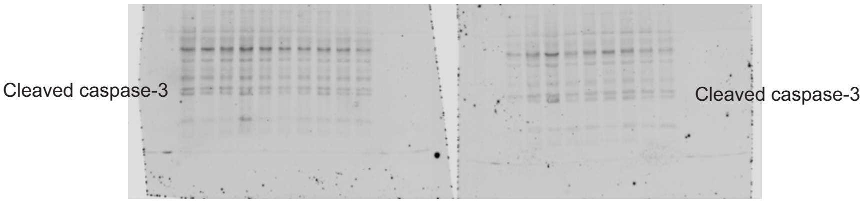

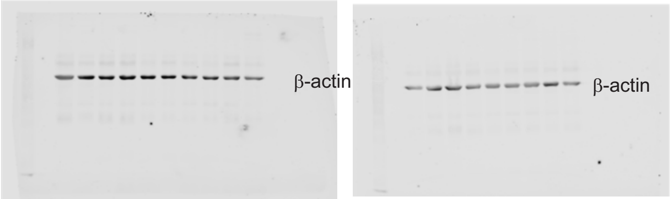

HO-1 gels

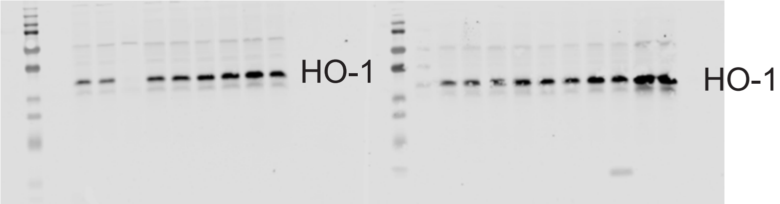

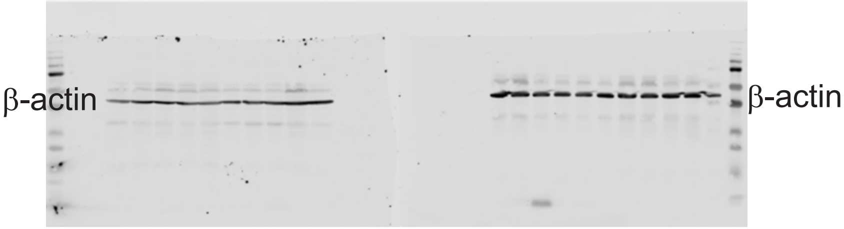

6.85 Gy

Ferritin gels

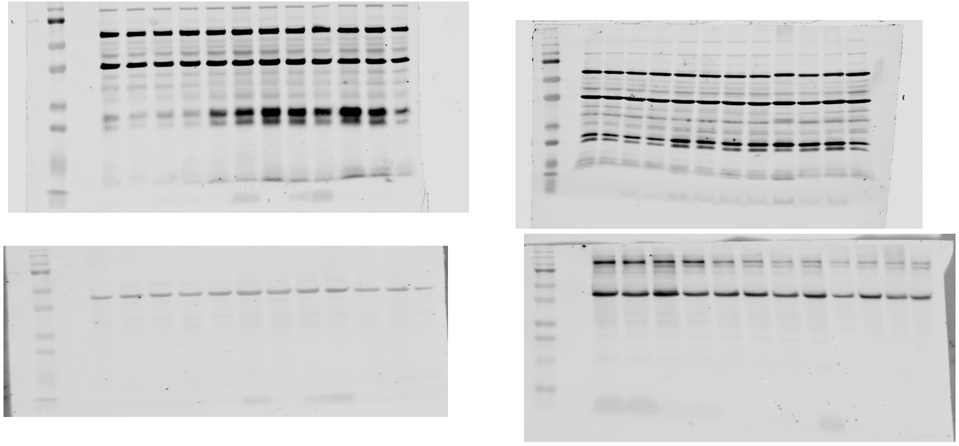

GPX-4 gels

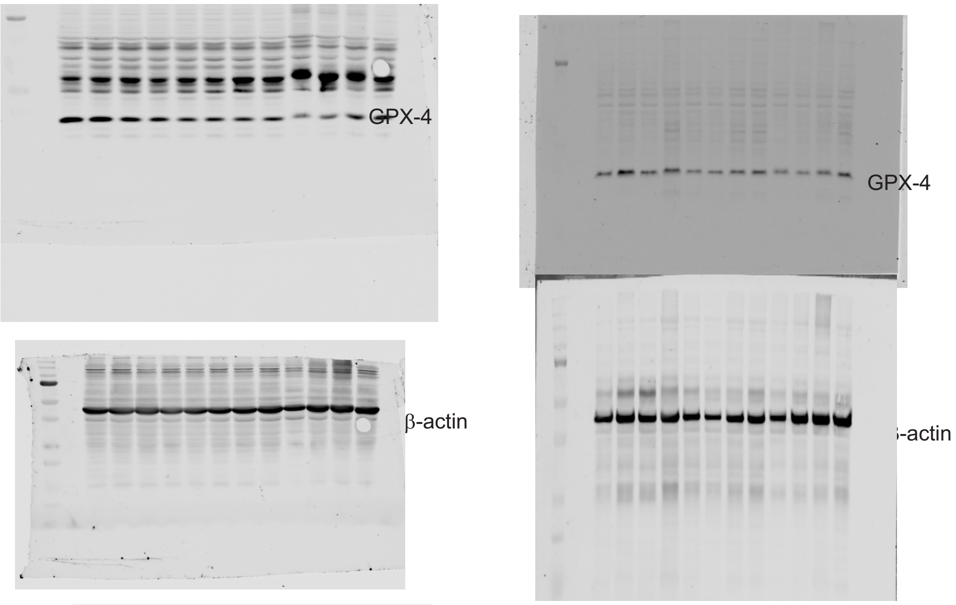

HO-1 gels

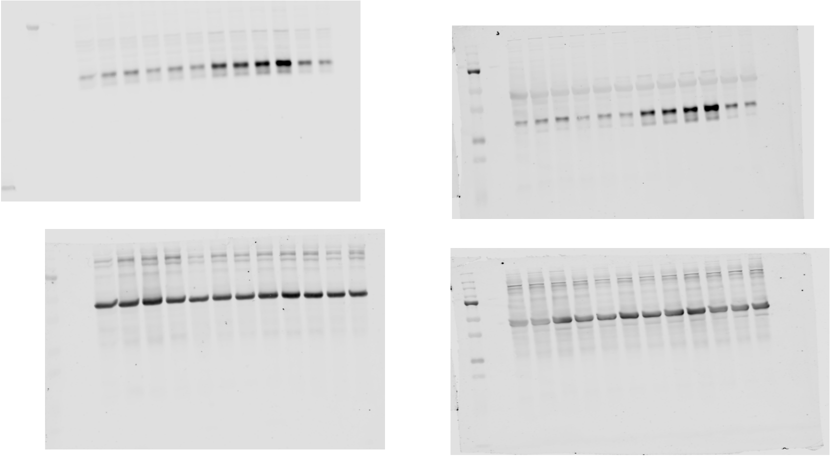

Caspase-3

Cleaved caspase-3

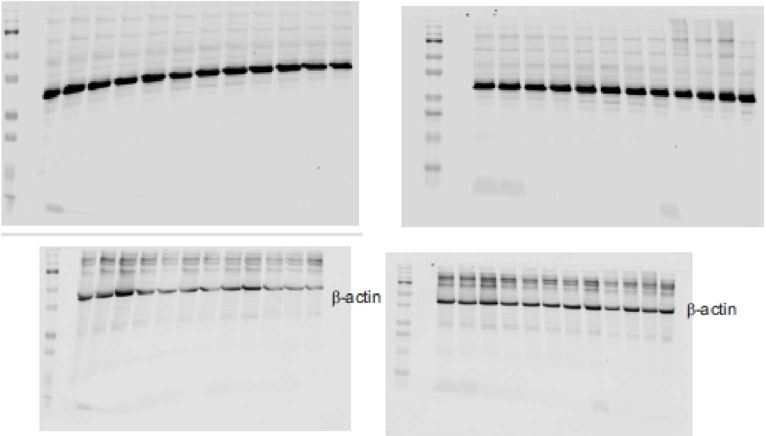

Cleaved caspase-3
