# Supplementary material for: Cell death and iron deposition in the liver in two murine models of acute radiation syndrome
Source: PLoS One. 2025 May 29;20(5):e0324361. doi: 10.1371/journal.pone.0324361 (PMC12121821; doi:10.1371/journal.pone.0324361)
Supplement: S3 Raw data file — Raw data for all qPCR, iron assays, and western blot data. (DOCX) [file pone.0324361.s003.docx]

Raw data files

qPCR Data from 7.9 Gy studies

| treatment | Fluor | Target | cDNA sample | AVERAGE CQ BY SAMPLE | AVERAGE GAPDH CQ BY SAMPLE | Normalize to GAPDH | Avg of controls | Normalized to control | Fold change in expresssion | LOG BASE 2 AVG EXPRESSION RESULTS FOR GRAPH | AVERAGE Normalized to CONTROL | FOLD CHANGE AVERAGE EXPRESSION | LOG BASE 2 AVG EXPRESSION RESULTS FOR GRAPH | StDev | SEM |
| --- | --- | --- | --- | --- | --- | --- | --- | --- | --- | --- | --- | --- | --- | --- | --- |
| SHAM CONTROL | SYBR | ITGAM | S1 | 33.30 | 20.57 | 12.73 | 12.73 | 0.00 | 1.00 | **0.00** | 0.00 | 1 | **0** | 0.32 | **0.16** |
|  | SYBR | ITGAM | S2 | 33.86 | 20.78 | 13.08 | 12.73266581 | 0.35 | 0.78 | **-0.35** |  |  |  |  |  |
|  | SYBR | ITGAM | S3 | 31.77 | 19.46 | 12.31 | 12.73266581 | -0.43 | 1.34 | **0.43** |  |  |  |  |  |
|  | SYBR | ITGAM | S4 | 32.30 | 19.49 | 12.81 | 12.73266581 | 0.08 | 0.95 | **-0.08** |  |  |  |  |  |
| Rad Veh 5 DPI | SYBR | ITGAM | RV5-1 | 32.22 | 20.20 | 12.02 | 12.73266581 | -0.71 | 1.63 | **0.71** | -0.94 | 1.92 | **0.94** | 0.61 | **0.31** |
|  | SYBR | ITGAM | RV5-2 | 31.15 | 19.48 | 11.67 | 12.73266581 | -1.06 | 2.09 | **1.06** |  |  |  |  |  |
|  | SYBR | ITGAM | RV5-3 | 29.64 | 18.63 | 11.01 | 12.73266581 | -1.72 | 3.29 | **1.72** |  |  |  |  |  |
|  | SYBR | ITGAM | RV5-4 | 31.41 | 18.94 | 12.47 | 12.73266581 | -0.27 | 1.20 | **0.27** |  |  |  |  |  |
| Rad Veh 12 DPI | SYBR | ITGAM | RV12-1 | 34.75 | 21.37 | 13.38 | 12.73266581 | 0.65 | 0.64 | **-0.65** | 0.29 | 0.82 | **-0.29** | 0.31 | **0.18** |
|  | SYBR | ITGAM | RV12-2 | 31.00 | 21.30 | 9.69 | 12.73266581 | -3.04 | 8.24 | **3.04** |  |  |  |  |  |
|  | SYBR | ITGAM | RV12-3 | 31.91 | 19.02 | 12.89 | 12.73266581 | 0.16 | 0.90 | **-0.16** |  |  |  |  |  |
|  | SYBR | ITGAM | RV12-4 | 32.15 | 19.34 | 12.81 | 12.73266581 | 0.07 | 0.95 | **-0.07** |  |  |  |  |  |
| Rad Veh 16 DPI | SYBR | ITGAM | RV16-1 | 31.47 | 18.43 | 13.04 | 12.73266581 | 0.31 | 0.81 | **-0.31** | 0.66 | 0.63 | **-0.66** | 0.47 | **0.24** |
|  | SYBR | ITGAM | RV16-2 | 31.82 | 18.59 | 13.22 | 12.73266581 | 0.49 | 0.71 | **-0.49** |  |  |  |  |  |
|  | SYBR | ITGAM | RV16-3 | 31.57 | 18.35 | 13.22 | 12.73266581 | 0.49 | 0.71 | **-0.49** |  |  |  |  |  |
|  | SYBR | ITGAM | RV16-4 | 35.71 | 21.62 | 14.09 | 12.73266581 | 1.36 | 0.39 | **-1.36** |  |  |  |  |  |
| Rad Veh 27 DPI | SYBR | ITGAM | RV27-2 | 30.68 | 18.17 | 12.50 | 12.73266581 | -0.23 | 1.17 | **0.23** | -0.11 | 1.08 | **0.11** | 0.10 | **0.06** |
|  | SYBR | ITGAM | RV27-3 | 30.99 | 18.30 | 12.69 | 12.73266581 | -0.04 | 1.03 | **0.04** |  |  |  |  |  |
|  | SYBR | ITGAM | RV27-4 | 31.27 | 18.60 | 12.67 | 12.73266581 | -0.06 | 1.04 | **0.06** |  |  |  |  |  |

| treatment | Fluor | Target | cDNA sample | AVERAGE CQ BY SAMPLE | AVERAGE GAPDH CQ BY SAMPLE | Normalize to GAPDH | Avg of controls | Normalized to control | Fold change in expresssion | LOG BASE 2 AVG EXPRESSION RESULTS FOR GRAPH | AVERAGE Normalized to CONTROL | FOLD CHANGE AVERAGE EXPRESSION | LOG BASE 2 AVG EXPRESSION RESULTS FOR GRAPH | StDev | SEM |
| --- | --- | --- | --- | --- | --- | --- | --- | --- | --- | --- | --- | --- | --- | --- | --- |
| SHAM CONTROL | SYBR | PTGS2 | S1 | 35.89 | 20.57 | 15.32 | 16.42 | -1.10 | 2.14 | **1.10** | 0.00 | 1 | **0** | 0.89 | **0.44** |
|  | SYBR | PTGS2 | S2 | 38.06 | 20.78 | 17.29 | 16.41917651 | 0.87 | 0.55 | **-0.87** |  |  |  |  |  |
|  | SYBR | PTGS2 | S3 | 36.43 | 19.46 | 16.97 | 16.41917651 | 0.55 | 0.68 | **-0.55** |  |  |  |  |  |
|  | SYBR | PTGS2 | S4 | 35.59 | 19.49 | 16.10 | 16.41917651 | -0.32 | 1.25 | **0.32** |  |  |  |  |  |
| Rad Veh 5 DPI | SYBR | PTGS2 | RV5-1 | 36.88 | 20.20 | 16.68 | 16.41917651 | 0.26 | 0.84 | **-0.26** | -0.36 | 1.28 | **0.36** | 0.59 | **0.34** |
|  | SYBR | PTGS2 | RV5-2 | 35.48 | 19.48 | 16.00 | 16.41917651 | -0.42 | 1.34 | **0.42** |  |  |  |  |  |
|  | SYBR | PTGS2 | RV5-3 | 28.47 | 18.63 | 9.84 | 16.41917651 | -6.58 | 95.75 | **6.58** |  |  |  |  |  |
|  | SYBR | PTGS2 | RV5-4 | 34.45 | 18.94 | 15.51 | 16.41917651 | -0.91 | 1.88 | **0.91** |  |  |  |  |  |
| Rad Veh 12 DPI | SYBR | PTGS2 | RV12-1 | 37.91 | 21.37 | 16.54 | 16.41917651 | 0.12 | 0.92 | **-0.12** | 0.01 | 0.99 | **-0.01** | 1.36 | **0.68** |
|  | SYBR | PTGS2 | RV12-2 | 35.90 | 21.30 | 14.59 | 16.41917651 | -1.83 | 3.55 | **1.83** |  |  |  |  |  |
|  | SYBR | PTGS2 | RV12-3 | 35.74 | 19.02 | 16.72 | 16.41917651 | 0.30 | 0.81 | **-0.30** |  |  |  |  |  |
|  | SYBR | PTGS2 | RV12-4 | 37.22 | 19.34 | 17.88 | 16.41917651 | 1.46 | 0.36 | **-1.46** |  |  |  |  |  |
| Rad Veh 16 DPI | SYBR | PTGS2 | RV16-1 | 36.20 | 18.43 | 17.77 | 16.41917651 | 1.36 | 0.39 | **-1.36** | 0.58 | 0.67 | **-0.58** | 1.29 | **0.65** |
|  | SYBR | PTGS2 | RV16-2 | 35.66 | 18.59 | 17.06 | 16.41917651 | 0.65 | 0.64 | **-0.65** |  |  |  |  |  |
|  | SYBR | PTGS2 | RV16-3 | 36.35 | 18.35 | 17.99 | 16.41917651 | 1.57 | 0.34 | **-1.57** |  |  |  |  |  |
|  | SYBR | PTGS2 | RV16-4 | 36.77 | 21.62 | 15.15 | 16.41917651 | -1.27 | 2.41 | **1.27** |  |  |  |  |  |
| Rad Veh 27 DPI | SYBR | PTGS2 | RV27-2 | 36.51 | 18.17 | 18.34 | 16.41917651 | 1.92 | 0.26 | **-1.92** | 1.89 | 0.27 | **-1.89** | 1.04 | **0.60** |
|  | SYBR | PTGS2 | RV27-3 | 35.56 | 18.30 | 17.26 | 16.41917651 | 0.84 | 0.56 | **-0.84** |  |  |  |  |  |
|  | SYBR | PTGS2 | RV27-4 | 37.94 | 18.60 | 19.34 | 16.41917651 | 2.92 | 0.13 | **-2.92** |  |  |  |  |  |

| treatment | Fluor | Target | cDNA sample | AVERAGE CQ BY SAMPLE | AVERAGE GAPDH CQ BY SAMPLE | Normalize to GAPDH | Avg of controls | Normalized to control | Fold change in expresssion | LOG BASE 2 AVG EXPRESSION RESULTS FOR GRAPH | AVERAGE Normalized to CONTROL | FOLD CHANGE AVERAGE EXPRESSION | LOG BASE 2 AVG EXPRESSION RESULTS FOR GRAPH | StDev | SEM |
| --- | --- | --- | --- | --- | --- | --- | --- | --- | --- | --- | --- | --- | --- | --- | --- |
| SHAM CONTROL | SYBR | NRF2 | S1 | 30.35 | 20.57 | 9.78 | 9.82 | -0.04 | 1.03 | **0.04** | 0.00 | 1 | **0** | 0.56 | **0.28** |
|  | SYBR | NRF2 | S2 | 29.82 | 20.78 | 9.04 | 9.821812427 | -0.78 | 1.72 | **0.78** |  |  |  |  |  |
|  | SYBR | NRF2 | S3 | 29.73 | 19.46 | 10.26 | 9.821812427 | 0.44 | 0.74 | **-0.44** |  |  |  |  |  |
|  | SYBR | NRF2 | S4 | 29.69 | 19.49 | 10.20 | 9.821812427 | 0.38 | 0.77 | **-0.38** |  |  |  |  |  |
| Rad Veh 5 DPI | SYBR | NRF2 | RV5-1 | 29.88 | 20.20 | 9.68 | 9.821812427 | -0.14 | 1.11 | **0.14** | -0.31 | 1.24 | **0.31** | 0.24 | **0.14** |
|  | SYBR | NRF2 | RV5-2 | 29.10 | 19.48 | 9.62 | 9.821812427 | -0.20 | 1.15 | **0.20** |  |  |  |  |  |
|  | SYBR | NRF2 | RV5-3 | 30.04 | 18.63 | 11.41 | 9.821812427 | 1.59 | 0.33 | **-1.59** |  |  |  |  |  |
|  | SYBR | NRF2 | RV5-4 | 28.18 | 18.94 | 9.24 | 9.821812427 | -0.59 | 1.50 | **0.59** |  |  |  |  |  |
| Rad Veh 12 DPI | SYBR | NRF2 | RV12-1 | 29.74 | 21.37 | 8.37 | 9.821812427 | -1.45 | 2.74 | **1.45** | -1.01 | 2.01 | **1.01** | 0.37 | **0.19** |
|  | SYBR | NRF2 | RV12-2 | 30.22 | 21.30 | 8.92 | 9.821812427 | -0.90 | 1.87 | **0.90** |  |  |  |  |  |
|  | SYBR | NRF2 | RV12-3 | 27.74 | 19.02 | 8.72 | 9.821812427 | -1.11 | 2.15 | **1.11** |  |  |  |  |  |
|  | SYBR | NRF2 | RV12-4 | 28.59 | 19.34 | 9.25 | 9.821812427 | -0.57 | 1.48 | **0.57** |  |  |  |  |  |
| Rad Veh 16 DPI | SYBR | NRF2 | RV16-1 | 29.26 | 18.43 | 10.83 | 9.821812427 | 1.01 | 0.50 | **-1.01** | 1.05 | 0.48 | **-1.05** | 0.27 | **0.13** |
|  |  | NRF2 | RV16-2 | 29.12 | 18.59 | 10.53 | 9.821812427 | 0.71 | 0.61 | **-0.71** |  |  |  |  |  |
|  |  | NRF2 | RV16-3 | 29.53 | 18.35 | 11.18 | 9.821812427 | 1.35 | 0.39 | **-1.35** |  |  |  |  |  |
|  | SYBR | NRF2 | RV16-4 | 32.57 | 21.62 | 10.95 | 9.821812427 | 1.13 | 0.46 | **-1.13** |  |  |  |  |  |
| Rad Veh 27 DPI | SYBR | NRF2 | RV27-2 | 29.35 | 18.17 | 11.18 | 9.821812427 | 1.35 | 0.39 | **-1.35** | 0.90 | 0.54 | **-0.90** | 0.68 | **0.39** |
|  | SYBR | NRF2 | RV27-3 | 29.34 | 18.30 | 11.04 | 9.821812427 | 1.22 | 0.43 | **-1.22** |  |  |  |  |  |
|  | SYBR | NRF2 | RV27-4 | 28.54 | 18.60 | 9.94 | 9.821812427 | 0.12 | 0.92 | **-0.12** |  |  |  |  |  |

| treatment | Fluor | Target | cDNA sample | AVERAGE CQ BY SAMPLE | AVERAGE GAPDH CQ BY SAMPLE | Normalize to GAPDH | Avg of controls | Normalized to control | Fold change in expresssion | LOG BASE 2 AVG EXPRESSION RESULTS FOR GRAPH | AVERAGE Normalized to CONTROL | FOLD CHANGE AVERAGE EXPRESSION | LOG BASE 2 AVG EXPRESSION RESULTS FOR GRAPH | StDev | SEM |
| --- | --- | --- | --- | --- | --- | --- | --- | --- | --- | --- | --- | --- | --- | --- | --- |
| SHAM CONTROL | SYBR | LCN-2 | S1 | 26.93 | 20.57 | 6.36 | 6.56 | -0.20 | 1.15 | **0.20** | 0.00 | 1 | **0** | 0.18 | **0.09** |
|  | SYBR | LCN-2 | S2 | 27.58 | 20.78 | 6.81 | 6.560274294 | 0.25 | 0.84 | **-0.25** |  |  |  |  |  |
|  | SYBR | LCN-2 | S3 | 26.03 | 19.46 | 6.56 | 6.560274294 | 0.00 | 1.00 | **0.00** |  |  |  |  |  |
|  | SYBR | LCN-2 | S4 | 26.00 | 19.49 | 6.51 | 6.560274294 | -0.05 | 1.04 | **0.05** |  |  |  |  |  |
| Rad Veh 5 DPI | SYBR | LCN-2 | RV5-1 | 27.02 | 20.20 | 6.82 | 6.560274294 | 0.26 | 0.84 | **-0.26** | 1.01 | 0.50 | **-1.01** | 1.47 | **0.74** |
|  | SYBR | LCN-2 | RV5-2 | 26.19 | 19.48 | 6.71 | 6.560274294 | 0.15 | 0.90 | **-0.15** |  |  |  |  |  |
|  | SYBR | LCN-2 | RV5-3 | 28.41 | 18.63 | 9.78 | 6.560274294 | 3.22 | 0.11 | **-3.22** |  |  |  |  |  |
|  | SYBR | LCN-2 | RV5-4 | 25.91 | 18.94 | 6.97 | 6.560274294 | 0.41 | 0.75 | **-0.41** |  |  |  |  |  |
| Rad Veh 12 DPI | SYBR | LCN-2 | RV12-1 | 27.19 | 21.37 | 5.82 | 6.560274294 | -0.74 | 1.67 | **0.74** | -2.87 | 7.32 | **2.87** | 3.48 | **1.74** |
|  | SYBR | LCN-2 | RV12-2 | 19.99 | 21.30 | -1.31 | 6.560274294 | -7.87 | 233.98 | **7.87** |  |  |  |  |  |
|  | SYBR | LCN-2 | RV12-3 | 25.28 | 19.02 | 6.26 | 6.560274294 | -0.30 | 1.23 | **0.30** |  |  |  |  |  |
|  | SYBR | LCN-2 | RV12-4 | 23.32 | 19.34 | 3.98 | 6.560274294 | -2.58 | 5.97 | **2.58** |  |  |  |  |  |
| Rad Veh 16 DPI | SYBR | LCN-2 | RV16-1 | 25.09 | 18.43 | 6.66 | 6.560274294 | 0.10 | 0.93 | **-0.10** | -0.23 | 1.17 | **0.23** | 0.34 | **0.17** |
|  | SYBR | LCN-2 | RV16-2 | 24.51 | 18.59 | 5.92 | 6.560274294 | -0.64 | 1.56 | **0.64** |  |  |  |  |  |
|  | SYBR | LCN-2 | RV16-3 | 24.92 | 18.35 | 6.56 | 6.560274294 | 0.00 | 1.00 | **0.00** |  |  |  |  |  |
|  | SYBR | LCN-2 | RV16-4 | 27.82 | 21.62 | 6.20 | 6.560274294 | -0.36 | 1.28 | **0.36** |  |  |  |  |  |
| Rad Veh 27 DPI | SYBR | LCN-2 | RV27-2 | 23.54 | 18.17 | 5.36 | 6.560274294 | -1.20 | 2.29 | **1.20** | -3.21 | 9.28 | **3.21** | 2.67 | **1.54** |
|  | SYBR | LCN-2 | RV27-3 | 22.66 | 18.30 | 4.36 | 6.560274294 | -2.20 | 4.59 | **2.20** |  |  |  |  |  |
|  | SYBR | LCN-2 | RV27-4 | 18.91 | 18.60 | 0.31 | 6.560274294 | -6.25 | 75.94 | **6.25** |  |  |  |  |  |

| treatment | Fluor | Target | cDNA sample | AVERAGE CQ BY SAMPLE | AVERAGE GAPDH CQ BY SAMPLE | Normalize to GAPDH | Avg of controls | Normalized to control | Fold change in expresssion | LOG BASE 2 AVG EXPRESSION RESULTS FOR GRAPH | AVERAGE Normalized to CONTROL | FOLD CHANGE AVERAGE EXPRESSION | LOG BASE 2 AVG EXPRESSION RESULTS FOR GRAPH | StDev | SEM |
| --- | --- | --- | --- | --- | --- | --- | --- | --- | --- | --- | --- | --- | --- | --- | --- |
| SHAM CONTROL | SYBR | FLVCR | S1 | 31.28 | 20.57 | 10.71 | 10.49 | 0.22 | 0.86 | **-0.22** | 0.00 | 1 | **0** | 0.22 | **0.11** |
|  | SYBR | FLVCR | S2 | 31.06 | 20.78 | 10.28 | 10.48742123 | -0.21 | 1.15 | **0.21** |  |  |  |  |  |
|  | SYBR | FLVCR | S3 | 30.10 | 19.46 | 10.64 | 10.48742123 | 0.15 | 0.90 | **-0.15** |  |  |  |  |  |
|  | SYBR | FLVCR | S4 | 29.81 | 19.49 | 10.32 | 10.48742123 | -0.16 | 1.12 | **0.16** |  |  |  |  |  |
| Rad Veh 5 DPI | SYBR | FLVCR | RV5-1 | 29.76 | 20.20 | 9.56 | 10.48742123 | -0.92 | 1.90 | **0.92** | -0.53 | 1.44 | **0.53** | 0.74 | **0.37** |
|  | SYBR | FLVCR | RV5-2 | 28.93 | 19.48 | 9.45 | 10.48742123 | -1.04 | 2.06 | **1.04** |  |  |  |  |  |
|  | SYBR | FLVCR | RV5-3 | 28.40 | 18.63 | 9.77 | 10.48742123 | -0.72 | 1.64 | **0.72** |  |  |  |  |  |
|  | SYBR | FLVCR | RV5-4 | 29.99 | 18.94 | 11.05 | 10.48742123 | 0.56 | 0.68 | **-0.56** |  |  |  |  |  |
| Rad Veh 12 DPI | SYBR | FLVCR | RV12-1 | 31.61 | 21.37 | 10.24 | 10.48742123 | -0.25 | 1.19 | **0.25** | -0.24 | 1.18 | **0.24** | 0.52 | **0.26** |
|  | SYBR | FLVCR | RV12-2 | 30.87 | 21.30 | 9.57 | 10.48742123 | -0.92 | 1.89 | **0.92** |  |  |  |  |  |
|  | SYBR | FLVCR | RV12-3 | 29.85 | 19.02 | 10.83 | 10.48742123 | 0.34 | 0.79 | **-0.34** |  |  |  |  |  |
|  | SYBR | FLVCR | RV12-4 | 29.70 | 19.34 | 10.36 | 10.48742123 | -0.12 | 1.09 | **0.12** |  |  |  |  |  |
| Rad Veh 16 DPI | SYBR | FLVCR | RV16-1 | 29.44 | 18.43 | 11.01 | 10.48742123 | 0.53 | 0.69 | **-0.53** | 0.80 | 0.57 | **-0.80** | 0.33 | **0.16** |
|  | SYBR | FLVCR | RV16-2 | 29.95 | 18.59 | 11.36 | 10.48742123 | 0.87 | 0.55 | **-0.87** |  |  |  |  |  |
|  | SYBR | FLVCR | RV16-3 | 29.42 | 18.35 | 11.06 | 10.48742123 | 0.58 | 0.67 | **-0.58** |  |  |  |  |  |
|  | SYBR | FLVCR | RV16-4 | 33.35 | 21.62 | 11.73 | 10.48742123 | 1.24 | 0.42 | **-1.24** |  |  |  |  |  |
| Rad Veh 27 DPI | SYBR | FLVCR | RV27-2 | 29.50 | 18.17 | 11.33 | 10.48742123 | 0.84 | 0.56 | **-0.84** | 0.73 | 0.60 | **-0.73** | 0.14 | **0.08** |
|  | SYBR | FLVCR | RV27-3 | 29.35 | 18.30 | 11.06 | 10.48742123 | 0.57 | 0.67 | **-0.57** |  |  |  |  |  |
|  | SYBR | FLVCR | RV27-4 | 29.87 | 18.60 | 11.27 | 10.48742123 | 0.78 | 0.58 | **-0.78** |  |  |  |  |  |

| treatment | Fluor | Target | cDNA sample | AVERAGE CQ BY SAMPLE | AVERAGE GAPDH CQ BY SAMPLE | Normalize to GAPDH | Avg of controls | Normalized to control | Fold change in expresssion | LOG BASE 2 AVG EXPRESSION RESULTS FOR GRAPH | AVERAGE Normalized to CONTROL | FOLD CHANGE AVERAGE EXPRESSION | LOG BASE 2 AVG EXPRESSION RESULTS FOR GRAPH | StDev | SEM |
| --- | --- | --- | --- | --- | --- | --- | --- | --- | --- | --- | --- | --- | --- | --- | --- |
| SHAM CONTROL | SYBR | SLC7A11 | S1 | 33.33 | 20.57 | 12.76 | 14.34 | -1.58 | 2.99 | **1.58** | 0.00 | 1 | **0** | 1.29 | **0.64** |
|  | SYBR | SLC7A11 | S2 | 34.61 | 20.78 | 13.83 | 14.34292124 | -0.51 | 1.42 | **0.51** |  |  |  |  |  |
|  | SYBR | SLC7A11 | S3 | 34.98 | 19.46 | 15.52 | 14.34292124 | 1.17 | 0.44 | **-1.17** |  |  |  |  |  |
|  | SYBR | SLC7A11 | S4 | 34.75 | 19.49 | 15.26 | 14.34292124 | 0.92 | 0.53 | **-0.92** |  |  |  |  |  |
| Rad Veh 5 DPI | SYBR | SLC7A11 | RV5-1 | 32.35 | 20.20 | 12.15 | 14.34292124 | -2.19 | 4.56 | **2.19** | -0.52 | 1.43 | **0.52** | 1.25 | **0.63** |
|  | SYBR | SLC7A11 | RV5-2 | 33.79 | 19.48 | 14.31 | 14.34292124 | -0.03 | 1.02 | **0.03** |  |  |  |  |  |
|  | SYBR | SLC7A11 | RV5-3 | 32.34 | 18.63 | 13.71 | 14.34292124 | -0.63 | 1.55 | **0.63** |  |  |  |  |  |
|  | SYBR | SLC7A11 | RV5-4 | 34.06 | 18.94 | 15.12 | 14.34292124 | 0.78 | 0.58 | **-0.78** |  |  |  |  |  |
| Rad Veh 12 DPI | SYBR | SLC7A11 | RV12-1 | 34.46 | 21.37 | 13.09 | 14.34292124 | -1.25 | 2.38 | **1.25** | -0.34 | 1.27 | **0.34** | 1.13 | **0.56** |
|  | SYBR | SLC7A11 | RV12-2 | 34.26 | 21.30 | 12.96 | 14.34292124 | -1.39 | 2.61 | **1.39** |  |  |  |  |  |
|  | SYBR | SLC7A11 | RV12-3 | 34.03 | 19.02 | 15.00 | 14.34292124 | 0.66 | 0.63 | **-0.66** |  |  |  |  |  |
|  | SYBR | SLC7A11 | RV12-4 | 34.29 | 19.34 | 14.95 | 14.34292124 | 0.60 | 0.66 | **-0.60** |  |  |  |  |  |
| Rad Veh 16 DPI | SYBR | SLC7A11 | RV16-1 | 34.63 | 18.43 | 16.20 | 14.34292124 | 1.86 | 0.28 | **-1.86** | 0.97 | 0.51 | **-0.97** | 1.39 | **0.69** |
|  | SYBR | SLC7A11 | RV16-2 | 34.21 | 18.59 | 15.62 | 14.34292124 | 1.28 | 0.41 | **-1.28** |  |  |  |  |  |
|  | SYBR | SLC7A11 | RV16-3 | 34.52 | 18.35 | 16.16 | 14.34292124 | 1.82 | 0.28 | **-1.82** |  |  |  |  |  |
|  | SYBR | SLC7A11 | RV16-4 | 34.89 | 21.62 | 13.27 | 14.34292124 | -1.07 | 2.10 | **1.07** |  |  |  |  |  |
| Rad Veh 27 DPI | SYBR | SLC7A11 | RV27-2 | 34.51 | 18.17 | 16.34 | 14.34292124 | 2.00 | 0.25 | **-2.00** | 1.42 | 0.37 | **-1.42** | 0.50 | **0.29** |
|  | SYBR | SLC7A11 | RV27-3 | 33.79 | 18.30 | 15.49 | 14.34292124 | 1.15 | 0.45 | **-1.15** |  |  |  |  |  |
|  | SYBR | SLC7A11 | RV27-4 | 34.06 | 18.60 | 15.46 | 14.34292124 | 1.12 | 0.46 | **-1.12** |  |  |  |  |  |

| treatment | Fluor | Target | cDNA sample | AVERAGE CQ BY SAMPLE | AVERAGE GAPDH CQ BY SAMPLE | Normalize to GAPDH | Avg of controls | Normalized to control | Fold change in expresssion | LOG BASE 2 AVG EXPRESSION RESULTS FOR GRAPH | AVERAGE Normalized to CONTROL | FOLD CHANGE AVERAGE EXPRESSION | LOG BASE 2 AVG EXPRESSION RESULTS FOR GRAPH | StDev | SEM |
| --- | --- | --- | --- | --- | --- | --- | --- | --- | --- | --- | --- | --- | --- | --- | --- |
| SHAM CONTROL | SYBR | GPX4 | S1 | 36.78 | 20.57 | 16.21 | 16.50 | -0.29 | 1.22 | **0.29** | 0.00 | 1 | **0** | 0.29 | **0.15** |
|  | SYBR | GPX4 | S2 | 37.09 | 20.78 | 16.31 | 16.50196225 | -0.19 | 1.14 | **0.19** |  |  |  |  |  |
|  | SYBR | GPX4 | S3 | 36.30 | 19.46 | 16.83 | 16.50196225 | 0.33 | 0.79 | **-0.33** |  |  |  |  |  |
|  | SYBR | GPX4 | S4 | 36.14 | 19.49 | 16.65 | 16.50196225 | 0.15 | 0.90 | **-0.15** |  |  |  |  |  |
| Rad Veh 5 DPI | SYBR | GPX4 | RV5-1 | 34.97 | 20.20 | 14.77 | 16.50196225 | -1.73 | 3.32 | **1.73** | -1.03 | 2.04 | **1.03** | 0.79 | **0.46** |
|  | SYBR | GPX4 | RV5-2 | 34.79 | 19.48 | 15.31 | 16.50196225 | -1.19 | 2.28 | **1.19** |  |  |  |  |  |
|  | SYBR | GPX4 | RV5-3 | 35.74 | 18.63 | 17.11 | 16.50196225 | 0.61 | 0.65 | **-0.61** |  |  |  |  |  |
|  | SYBR | GPX4 | RV5-4 | 35.28 | 18.94 | 16.33 | 16.50196225 | -0.17 | 1.12 | **0.17** |  |  |  |  |  |
| Rad Veh 12 DPI | SYBR | GPX4 | RV12-1 | 38.14 | 21.37 | 16.77 | 16.50196225 | 0.26 | 0.83 | **-0.26** | -0.40 | 1.32 | **0.40** | 0.90 | **0.45** |
|  | SYBR | GPX4 | RV12-2 | 38.27 | 21.30 | 16.96 | 16.50196225 | 0.46 | 0.73 | **-0.46** |  |  |  |  |  |
|  | SYBR | GPX4 | RV12-3 | 34.13 | 19.02 | 15.11 | 16.50196225 | -1.40 | 2.63 | **1.40** |  |  |  |  |  |
|  | SYBR | GPX4 | RV12-4 | 34.92 | 19.34 | 15.58 | 16.50196225 | -0.92 | 1.90 | **0.92** |  |  |  |  |  |
| Rad Veh 16 DPI | SYBR | GPX4 | RV16-1 | 35.56 | 18.43 | 17.13 | 16.50196225 | 0.63 | 0.64 | **-0.63** | 1.03 | 0.49 | **-1.03** | 0.31 | **0.15** |
|  | SYBR | GPX4 | RV16-2 | 36.41 | 18.59 | 17.81 | 16.50196225 | 1.31 | 0.40 | **-1.31** |  |  |  |  |  |
|  | SYBR | GPX4 | RV16-3 | 35.80 | 18.35 | 17.45 | 16.50196225 | 0.95 | 0.52 | **-0.95** |  |  |  |  |  |
|  | SYBR | GPX4 | RV16-4 | 39.35 | 21.62 | 17.73 | 16.50196225 | 1.23 | 0.43 | **-1.23** |  |  |  |  |  |
| Rad Veh 27 DPI | SYBR | GPX4 | RV27-2 | 35.24 | 18.17 | 17.07 | 16.50196225 | 0.56 | 0.68 | **-0.56** | 0.81 | 0.57 | **-0.81** | 0.34 | **0.20** |
|  | SYBR | GPX4 | RV27-3 | 36.00 | 18.30 | 17.70 | 16.50196225 | 1.20 | 0.44 | **-1.20** |  |  |  |  |  |
|  | SYBR | GPX4 | RV27-4 | 35.78 | 18.60 | 17.18 | 16.50196225 | 0.68 | 0.62 | **-0.68** |  |  |  |  |  |

| treatment | Fluor | Target | cDNA sample | AVERAGE CQ BY SAMPLE | AVERAGE GAPDH CQ BY SAMPLE | Normalize to GAPDH | Avg of controls | Normalized to control | Fold change in expresssion | LOG BASE 2 AVG EXPRESSION RESULTS FOR GRAPH | AVERAGE Normalized to CONTROL | FOLD CHANGE AVERAGE EXPRESSION | LOG BASE 2 AVG EXPRESSION RESULTS FOR GRAPH | StDev | SEM |
| --- | --- | --- | --- | --- | --- | --- | --- | --- | --- | --- | --- | --- | --- | --- | --- |
| SHAM CONTROL | SYBR | TRF | S1 | 24.48 | 20.57 | 3.91 | 3.65 | 0.26 | 0.83 | **-0.26** | 0.00 | 1 | **0** | 0.19 | **0.10** |
|  | SYBR | TRF | S2 | 24.23 | 20.78 | 3.45 | 3.645370874 | -0.19 | 1.14 | **0.19** |  |  |  |  |  |
|  | SYBR | TRF | S3 | 23.08 | 19.46 | 3.61 | 3.645370874 | -0.03 | 1.02 | **0.03** |  |  |  |  |  |
|  | SYBR | TRF | S4 | 23.09 | 19.49 | 3.61 | 3.645370874 | -0.04 | 1.03 | **0.04** |  |  |  |  |  |
| Rad Veh 5 DPI | SYBR | TRF | RV5-1 | 21.91 | 20.20 | 1.71 | 3.645370874 | -1.94 | 3.83 | **1.94** | -1.87 | 3.66 | **1.87** | 0.16 | **0.09** |
|  | SYBR | TRF | RV5-2 | 21.14 | 19.48 | 1.66 | 3.645370874 | -1.99 | 3.97 | **1.99** |  |  |  |  |  |
|  | SYBR | TRF | RV5-3 | 28.97 | 18.63 | 10.34 | 3.645370874 | 6.69 | 0.01 | **-6.69** |  |  |  |  |  |
|  | SYBR | TRF | RV5-4 | 20.89 | 18.94 | 1.95 | 3.645370874 | -1.69 | 3.24 | **1.69** |  |  |  |  |  |
| Rad Veh 12 DPI | SYBR | TRF | RV12-1 | 24.71 | 21.37 | 3.34 | 3.645370874 | -0.30 | 1.23 | **0.30** | -0.47 | 1.38 | **0.47** | 0.47 | **0.23** |
|  | SYBR | TRF | RV12-2 | 23.81 | 21.30 | 2.51 | 3.645370874 | -1.14 | 2.20 | **1.14** |  |  |  |  |  |
|  | SYBR | TRF | RV12-3 | 22.29 | 19.02 | 3.27 | 3.645370874 | -0.37 | 1.30 | **0.37** |  |  |  |  |  |
|  | SYBR | TRF | RV12-4 | 22.93 | 19.34 | 3.59 | 3.645370874 | -0.06 | 1.04 | **0.06** |  |  |  |  |  |
| Rad Veh 16 DPI | SYBR | TRF | RV16-1 | 21.27 | 18.43 | 2.84 | 3.645370874 | -0.81 | 1.75 | **0.81** | -0.13 | 1.10 | **0.13** | 0.53 | **0.27** |
|  | SYBR | TRF | RV16-2 | 22.16 | 18.59 | 3.56 | 3.645370874 | -0.08 | 1.06 | **0.08** |  |  |  |  |  |
|  | SYBR | TRF | RV16-3 | 21.85 | 18.35 | 3.50 | 3.645370874 | -0.14 | 1.10 | **0.14** |  |  |  |  |  |
|  | SYBR | TRF | RV16-4 | 25.77 | 21.62 | 4.15 | 3.645370874 | 0.50 | 0.71 | **-0.50** |  |  |  |  |  |
| Rad Veh 27 DPI | SYBR | TRF | RV27-2 | 22.24 | 18.17 | 4.07 | 3.645370874 | 0.42 | 0.75 | **-0.42** | 0.63 | 0.65 | **-0.63** | 0.42 | **0.24** |
|  | SYBR | TRF | RV27-3 | 22.29 | 18.30 | 3.99 | 3.645370874 | 0.35 | 0.79 | **-0.35** |  |  |  |  |  |
|  | SYBR | TRF | RV27-4 | 23.36 | 18.60 | 4.76 | 3.645370874 | 1.11 | 0.46 | **-1.11** |  |  |  |  |  |

| treatment | Fluor | Target | cDNA sample | AVERAGE CQ BY SAMPLE | AVERAGE GAPDH CQ BY SAMPLE | Normalize to GAPDH | Avg of controls | Normalized to control | Fold change in expresssion | LOG BASE 2 AVG EXPRESSION RESULTS FOR GRAPH | AVERAGE Normalized to CONTROL | FOLD CHANGE AVERAGE EXPRESSION | LOG BASE 2 AVG EXPRESSION RESULTS FOR GRAPH | StDev | SEM |
| --- | --- | --- | --- | --- | --- | --- | --- | --- | --- | --- | --- | --- | --- | --- | --- |
| SHAM CONTROL | SYBR | TFR1 | S1 | 27.95 | 20.57 | 7.37 | 7.43 | -0.05 | 1.04 | **0.05** | 0.00 | 1 | **0** | 0.63 | **0.36** |
|  | SYBR | TFR1 | S2 | 27.61 | 20.78 | 6.83 | 7.429554493 | -0.60 | 1.52 | **0.60** |  |  |  |  |  |
|  | SYBR | TFR1 | S3 | 27.55 | 19.46 | 8.09 | 7.429554493 | 0.66 | 0.63 | **-0.66** |  |  |  |  |  |
|  | SYBR | TFR1 | S4 | 28.26 | 19.49 | 8.77 | 7.429554493 | 1.34 | 0.39 | **-1.34** |  |  |  |  |  |
| Rad Veh 5 DPI | SYBR | TFR1 | RV5-1 | 29.41 | 20.20 | 9.21 | 7.429554493 | 1.78 | 0.29 | **-1.78** | 1.05 | 0.48 | **-1.05** | 0.63 | **0.37** |
|  | SYBR | TFR1 | RV5-2 | 27.62 | 19.48 | 8.14 | 7.429554493 | 0.71 | 0.61 | **-0.71** |  |  |  |  |  |
|  | SYBR | TFR1 | RV5-3 | 23.32 | 18.63 | 4.69 | 7.429554493 | -2.74 | 6.68 | **2.74** |  |  |  |  |  |
|  | SYBR | TFR1 | RV5-4 | 27.02 | 18.94 | 8.08 | 7.429554493 | 0.65 | 0.64 | **-0.65** |  |  |  |  |  |
| Rad Veh 12 DPI | SYBR | TFR1 | RV12-1 | 28.86 | 21.37 | 7.49 | 7.429554493 | 0.06 | 0.96 | **-0.06** | 0.16 | 0.90 | **-0.16** | 0.41 | **0.20** |
|  | SYBR | TFR1 | RV12-2 | 28.84 | 21.30 | 7.54 | 7.429554493 | 0.11 | 0.93 | **-0.11** |  |  |  |  |  |
|  | SYBR | TFR1 | RV12-3 | 27.17 | 19.02 | 8.15 | 7.429554493 | 0.72 | 0.61 | **-0.72** |  |  |  |  |  |
|  | SYBR | TFR1 | RV12-4 | 26.50 | 19.34 | 7.17 | 7.429554493 | -0.26 | 1.20 | **0.26** |  |  |  |  |  |
| Rad Veh 16 DPI | SYBR | TFR1 | RV16-1 | 27.19 | 18.43 | 8.77 | 7.429554493 | 1.34 | 0.40 | **-1.34** | 1.35 | 0.39 | **-1.35** | 0.02 | **0.01** |
|  | SYBR | TFR1 | RV16-2 | 27.39 | 18.59 | 8.79 | 7.429554493 | 1.36 | 0.39 | **-1.36** |  |  |  |  |  |
|  | SYBR | TFR1 | RV16-3 | 27.15 | 18.35 | 8.79 | 7.429554493 | 1.36 | 0.39 | **-1.36** |  |  |  |  |  |
|  | SYBR | TFR1 | RV16-4 | 30.38 | 21.62 | 8.76 | 7.429554493 | 1.33 | 0.40 | **-1.33** |  |  |  |  |  |
| Rad Veh 27 DPI | SYBR | TFR1 | RV27-2 | 26.16 | 18.17 | 7.98 | 7.429554493 | 0.55 | 0.68 | **-0.55** | 1.09 | 0.47 | **-1.09** | 0.50 | **0.29** |
|  | SYBR | TFR1 | RV27-3 | 26.92 | 18.30 | 8.62 | 7.429554493 | 1.19 | 0.44 | **-1.19** |  |  |  |  |  |
|  | SYBR | TFR1 | RV27-4 | 27.57 | 18.60 | 8.97 | 7.429554493 | 1.54 | 0.34 | **-1.54** |  |  |  |  |  |

| treatment | Fluor | Target | cDNA sample | AVERAGE CQ BY SAMPLE | AVERAGE GAPDH CQ BY SAMPLE | Normalize to GAPDH | Avg of controls | Normalized to control | Fold change in expresssion | LOG BASE 2 AVG EXPRESSION RESULTS FOR GRAPH | AVERAGE Normalized to CONTROL | FOLD CHANGE AVERAGE EXPRESSION | LOG BASE 2 AVG EXPRESSION RESULTS FOR GRAPH | StDev | SEM |
| --- | --- | --- | --- | --- | --- | --- | --- | --- | --- | --- | --- | --- | --- | --- | --- |
| SHAM CONTROL | SYBR | SLC40A1 | S1 | 26.25 | 20.57 | 5.68 | 5.49 | 0.19 | 0.88 | **-0.19** | 0.00 | 1 | **0** | 0.14 | **0.07** |
|  | SYBR | SLC40A1 | S2 | 26.27 | 20.78 | 5.49 | 5.48865068 | 0.00 | 1.00 | **0.00** |  |  |  |  |  |
|  | SYBR | SLC40A1 | S3 | 24.86 | 19.46 | 5.39 | 5.48865068 | -0.10 | 1.07 | **0.10** |  |  |  |  |  |
|  | SYBR | SLC40A1 | S4 | 24.87 | 19.49 | 5.39 | 5.48865068 | -0.10 | 1.07 | **0.10** |  |  |  |  |  |
| Rad Veh 5 DPI | SYBR | SLC40A1 | RV5-1 | 23.91 | 20.20 | 3.71 | 5.48865068 | -1.78 | 3.43 | **1.78** | -1.73 | 3.31 | **1.73** | 0.19 | **0.11** |
|  | SYBR | SLC40A1 | RV5-2 | 23.09 | 19.48 | 3.61 | 5.48865068 | -1.88 | 3.68 | **1.88** |  |  |  |  |  |
|  | SYBR | SLC40A1 | RV5-3 | 25.20 | 18.63 | 6.57 | 5.48865068 | 1.08 | 0.47 | **-1.08** |  |  |  |  |  |
|  | SYBR | SLC40A1 | RV5-4 | 22.92 | 18.94 | 3.97 | 5.48865068 | -1.52 | 2.86 | **1.52** |  |  |  |  |  |
| Rad Veh 12 DPI | SYBR | SLC40A1 | RV12-1 | 26.47 | 21.37 | 5.10 | 5.48865068 | -0.39 | 1.31 | **0.39** | -0.25 | 1.19 | **0.25** | 0.29 | **0.15** |
|  | SYBR | SLC40A1 | RV12-2 | 26.26 | 21.30 | 4.96 | 5.48865068 | -0.53 | 1.45 | **0.53** |  |  |  |  |  |
|  | SYBR | SLC40A1 | RV12-3 | 24.28 | 19.02 | 5.26 | 5.48865068 | -0.23 | 1.18 | **0.23** |  |  |  |  |  |
|  | SYBR | SLC40A1 | RV12-4 | 24.98 | 19.34 | 5.64 | 5.48865068 | 0.15 | 0.90 | **-0.15** |  |  |  |  |  |
| Rad Veh 16 DPI | SYBR | SLC40A1 | RV16-1 | 23.44 | 18.43 | 5.01 | 5.48865068 | -0.48 | 1.40 | **0.48** | -0.01 | 1.01 | **0.01** | 0.48 | **0.24** |
|  | SYBR | SLC40A1 | RV16-2 | 23.88 | 18.59 | 5.28 | 5.48865068 | -0.21 | 1.16 | **0.21** |  |  |  |  |  |
|  | SYBR | SLC40A1 | RV16-3 | 23.84 | 18.35 | 5.49 | 5.48865068 | 0.00 | 1.00 | **0.00** |  |  |  |  |  |
|  | SYBR | SLC40A1 | RV16-4 | 27.75 | 21.62 | 6.13 | 5.48865068 | 0.64 | 0.64 | **-0.64** |  |  |  |  |  |
| Rad Veh 27 DPI | SYBR | SLC40A1 | RV27-2 | 24.19 | 18.17 | 6.02 | 5.48865068 | 0.53 | 0.69 | **-0.53** | 0.56 | 0.68 | **-0.56** | 0.61 | **0.35** |
|  | SYBR | SLC40A1 | RV27-3 | 23.75 | 18.30 | 5.45 | 5.48865068 | -0.04 | 1.03 | **0.04** |  |  |  |  |  |
|  | SYBR | SLC40A1 | RV27-4 | 25.28 | 18.60 | 6.68 | 5.48865068 | 1.19 | 0.44 | **-1.19** |  |  |  |  |  |

| treatment | Fluor | Target | cDNA sample | AVERAGE CQ BY SAMPLE | AVERAGE GAPDH CQ BY SAMPLE | Normalize to GAPDH | Avg of controls | Normalized to control | Fold change in expresssion | LOG BASE 2 AVG EXPRESSION RESULTS FOR GRAPH | AVERAGE Normalized to CONTROL | FOLD CHANGE AVERAGE EXPRESSION | LOG BASE 2 AVG EXPRESSION RESULTS FOR GRAPH | StDev | SEM |
| --- | --- | --- | --- | --- | --- | --- | --- | --- | --- | --- | --- | --- | --- | --- | --- |
| SHAM CONTROL | SYBR | HO-1 | S1 | 28.24 | 20.57 | 7.67 | 7.26 | 0.41 | 0.75 | **-0.41** | 0.00 | 1 | **0** | 0.49 | **0.24** |
|  | SYBR | HO-1 | S2 | 28.45 | 20.78 | 7.68 | 7.259224992 | 0.42 | 0.75 | **-0.42** |  |  |  |  |  |
|  | SYBR | HO-1 | S3 | 26.19 | 19.46 | 6.73 | 7.259224992 | -0.53 | 1.45 | **0.53** |  |  |  |  |  |
|  | SYBR | HO-1 | S4 | 26.45 | 19.49 | 6.97 | 7.259224992 | -0.29 | 1.23 | **0.29** |  |  |  |  |  |
| Rad Veh 5 DPI | SYBR | HO-1 | RV5-1 | 26.44 | 20.20 | 6.24 | 7.259224992 | -1.01 | 2.02 | **1.01** | -1.13 | 2.19 | **1.13** | 0.15 | **0.09** |
|  | SYBR | HO-1 | RV5-2 | 25.67 | 19.48 | 6.19 | 7.259224992 | -1.07 | 2.10 | **1.07** |  |  |  |  |  |
|  | SYBR | HO-1 | RV5-3 | 27.10 | 18.63 | 8.47 | 7.259224992 | 1.21 | 0.43 | **-1.21** |  |  |  |  |  |
|  | SYBR | HO-1 | RV5-4 | 24.90 | 18.94 | 5.96 | 7.259224992 | -1.30 | 2.47 | **1.30** |  |  |  |  |  |
| Rad Veh 12 DPI | SYBR | HO-1 | RV12-1 | 28.69 | 21.37 | 7.32 | 7.259224992 | 0.06 | 0.96 | **-0.06** | -1.35 | 2.55 | **1.35** | 0.94 | **0.47** |
|  | SYBR | HO-1 | RV12-2 | 26.71 | 21.30 | 5.41 | 7.259224992 | -1.85 | 3.61 | **1.85** |  |  |  |  |  |
|  | SYBR | HO-1 | RV12-3 | 24.53 | 19.02 | 5.51 | 7.259224992 | -1.75 | 3.37 | **1.75** |  |  |  |  |  |
|  | SYBR | HO-1 | RV12-4 | 24.74 | 19.34 | 5.41 | 7.259224992 | -1.85 | 3.61 | **1.85** |  |  |  |  |  |
| Rad Veh 16 DPI | SYBR | HO-1 | RV16-1 | 23.35 | 18.43 | 4.92 | 7.259224992 | -2.34 | 5.06 | **2.34** | -1.77 | 3.40 | **1.77** | 0.74 | **0.37** |
|  | SYBR | HO-1 | RV16-2 | 24.12 | 18.59 | 5.53 | 7.259224992 | -1.73 | 3.32 | **1.73** |  |  |  |  |  |
|  | SYBR | HO-1 | RV16-3 | 23.35 | 18.35 | 5.00 | 7.259224992 | -2.26 | 4.79 | **2.26** |  |  |  |  |  |
|  | SYBR | HO-1 | RV16-4 | 28.14 | 21.62 | 6.52 | 7.259224992 | -0.74 | 1.67 | **0.74** |  |  |  |  |  |
| Rad Veh 27 DPI | SYBR | HO-1 | RV27-2 | 23.14 | 18.17 | 4.96 | 7.259224992 | -2.30 | 4.91 | **2.30** | -2.12 | 4.35 | **2.12** | 0.22 | **0.13** |
|  | SYBR | HO-1 | RV27-3 | 23.36 | 18.30 | 5.06 | 7.259224992 | -2.20 | 4.59 | **2.20** |  |  |  |  |  |
|  | SYBR | HO-1 | RV27-4 | 23.99 | 18.60 | 5.39 | 7.259224992 | -1.87 | 3.65 | **1.87** |  |  |  |  |  |

Data processed from raw data from Bio-Rad qPCR machine 6.85 Gy

IL-1beta

| Sham | 10 | 17 | 24 |
| --- | --- | --- | --- |
| 1.01 | 0.14 | 0.59 | 0.66 |
| 0.94 | 0.23 | 0.66 | 0.68 |
| 1.05 | 0.16 | 0.42 | 0.74 |
|  | 0.34 | 0.39 | 0.54 |

SAA

| sham | 7 | 14 | 21 |
| --- | --- | --- | --- |
| 0.77 | 1.18 | 3.92 | 2.2 |
| 1.04 | 1.15 | 3.0 | 3.7 |
| 1.25 | 0.89 | 3.2 | 1.92 |
|  | 0.75 | 3.51 | 0.94 |
|  | 1.83 |  |  |

qPCR data from 6.85 Gy studies

| treatment | Fluor | Target | cDNA sample | AVERAGE CQ BY SAMPLE | AVERAGE GAPDH CQ BY SAMPLE | Normalize to GAPDH | Avg of controls | Normalized to control | Fold change in expresssion | LOG BASE 2 AVG EXPRESSION RESULTS FOR GRAPH | AVERAGE Normalized to CONTROL | FOLD CHANGE AVERAGE EXPRESSION | LOG BASE 2 AVG EXPRESSION RESULTS FOR GRAPH | StDev | SEM |
| --- | --- | --- | --- | --- | --- | --- | --- | --- | --- | --- | --- | --- | --- | --- | --- |
| SHAM CONTROL | SYBR | FLVCR1 | SHAM-1 | 29.15 | 19.63 | 9.52 | 9.8275 | -0.3075 | 1.23756131 | **0.3075** | 0 | 1 | **0** | 0.44 | **0.22** |
|  | SYBR | FLVCR1 | SHAM-2 | 29.72 | 19.77 | 9.95 | 9.8275 | 0.1225 | 0.9185944677 | **-0.1225** |  |  |  |  |  |
|  | SYBR | FLVCR1 | SHAM-3 | 29.2 | 19.3 | 9.9 | 9.8275 | 0.0725 | 0.9509886317 | **-0.0725** |  |  |  |  |  |
|  | SYBR | FLVCR1 | SHAM-4 | 29.24 | 19.3 | 9.94 | 9.8275 | 0.1125 | 0.9249837975 | **-0.1125** |  |  |  |  |  |
| RV 7 dpi | SYBR | FLVCR1 | RV7-1 | 28.91 | 19.07 | 9.84 | 9.8275 | 0.0125 | 0.9913730875 | **-0.0125** | 0.0125 | 0.9913730875 | **-0.0125** | 1.18 | **0.59** |
|  | SYBR | FLVCR1 | RV7-2 | 28.55 | 19.11 | 9.44 | 9.8275 | -0.3875 | 1.308124631 | **0.3875** |  |  |  |  |  |
|  | SYBR | FLVCR1 | RV7-3 | 28.86 | 18.9 | 9.96 | 9.8275 | 0.1325 | 0.9122492722 | **-0.1325** |  |  |  |  |  |
|  | SYBR | FLVCR1 | RV7-4 | 28.8 | 18.68 | 10.12 | 9.8275 | 0.2925 | 0.8164859696 | **-0.2925** |  |  |  |  |  |
| RV 14 dpi | SYBR | FLVCR1 | RV14-1 | 29.4 | 19.3 | 10.1 | 9.8275 | 0.2725 | 0.827883689 | **-0.2725** | 0.1475 | 0.9028135646 | **-0.1475** | 1.18 | **0.59** |
|  | SYBR | FLVCR1 | RV14-2 | 28.25 | 18.28 | 9.97 | 9.8275 | 0.1425 | 0.9059479062 | **-0.1425** |  |  |  |  |  |
|  | SYBR | FLVCR1 | RV14-3 | 28.71 | 18.97 | 9.74 | 9.8275 | -0.0875 | 1.062527367 | **0.0875** |  |  |  |  |  |
|  | SYBR | FLVCR1 | RV14-4 | 29.05 | 18.96 | 10.09 | 9.8275 | 0.2625 | 0.8336420754 | **-0.2625** |  |  |  |  |  |
| RV 21 dpi | SYBR | FLVCR1 | RV21-1 | 28.41 | 18.79 | 9.62 | 9.8275 | -0.2075 | 1.154685532 | **0.2075** | 0.0175 | 0.9879431971 | **-0.0175** | 0.64 | **0.37** |
|  | SYBR | FLVCR1 | RV21-2 | 28.75 | 18.66 | 10.09 | 9.8275 | 0.2625 | 0.8336420754 | **-0.2625** |  |  |  |  |  |
|  | SYBR | FLVCR1 | RV21-3 | 28.73 | 18.72 | 10.01 | 9.8275 | 0.1825 | 0.8811747131 | **-0.1825** |  |  |  |  |  |
|  | SYBR | FLVCR1 | RV21-4 | 28.54 | 18.88 | 9.66 | 9.8275 | -0.1675 | 1.123110595 | **0.1675** |  |  |  |  |  |
| RV 28dpi | SYBR | FLVCR1 | RV28-1 | 29.33 | 19.3 | 10.03 | 9.8275 | 0.2025 | 0.8690433204 | **-0.2025** | 0.0275 | 0.981118975 | **-0.0275** | 1.3 | **0.75** |
|  | SYBR | FLVCR1 | RV28-2 | 29.12 | 19.43 | 9.69 | 9.8275 | -0.1375 | 1.099997313 | **0.1375** |  |  |  |  |  |
|  | SYBR | FLVCR1 | RV28-3 | 29.09 | 19.42 | 9.67 | 9.8275 | -0.1575 | 1.115352704 | **0.1575** |  |  |  |  |  |
|  | SYBR | FLVCR1 | RV28-4 | 29.77 | 19.74 | 10.03 | 9.8275 | 0.2025 | 0.8690433204 | **-0.2025** |  |  |  |  |  |

| treatment |  | Fluor | Target | cDNA sample | AVERAGE CQ BY SAMPLE | AVERAGE GAPDH CQ BY SAMPLE | Normalize to GAPDH | Avg of controls | Normalized to control | Fold change in expresssion | LOG BASE 2 AVG EXPRESSION RESULTS FOR GRAPH | AVERAGE Normalized to CONTROL | FOLD CHANGE AVERAGE EXPRESSION | LOG BASE 2 AVG EXPRESSION RESULTS FOR GRAPH | StDev | SEM |
| --- | --- | --- | --- | --- | --- | --- | --- | --- | --- | --- | --- | --- | --- | --- | --- | --- |
| SHAM CONTROL |  | SYBR | TFRI | SHAM-1 | 28.97 | 19.63 | 9.34 | 9.54 | -0.2 | 1.148698355 | **0.2** | 0 | 1 | **0** | 0.44 | **0.22** |
|  |  | SYBR | TFRI | SHAM-2 | 29.77 | 19.77 | 10 | 9.54 | 0.46 | 0.7269862587 | **-0.46** |  |  |  |  |  |
|  |  | SYBR | TFRI | SHAM-3 | 28.6 | 19.3 | 9.3 | 9.54 | -0.24 | 1.180992661 | **0.24** |  |  |  |  |  |
|  |  | SYBR | TFRI | SHAM-4 | 28.82 | 19.3 | 9.52 | 9.54 | -0.02 | 1.01395948 | **0.02** |  |  |  |  |  |
| RV 7 dpi |  | SYBR | TFRI | RV7-1 | 27.95 | 19.07 | 8.88 | 9.54 | -0.66 | 1.580082624 | **0.66** | -0.9 | 1.866065983 | **0.9** | 1.18 | **0.59** |
|  |  | SYBR | TFRI | RV7-2 | 27.66 | 19.11 | 8.55 | 9.54 | -0.99 | 1.986184991 | **0.99** |  |  |  |  |  |
|  |  | SYBR | TFRI | RV7-3 | 27.61 | 18.9 | 8.71 | 9.54 | -0.83 | 1.777685362 | **0.83** |  |  |  |  |  |
|  |  | SYBR | TFRI | RV7-4 | 27.1 | 18.68 | 8.42 | 9.54 | -1.12 | 2.173469725 | **1.12** |  |  |  |  |  |
| RV 14 dpi |  | SYBR | TFRI | RV14-1 | 27.8 | 19.3 | 8.5 | 9.54 | -1.04 | 2.056227653 | **1.04** | -0.9925 | 1.989629771 | **0.9925** | 1.18 | **0.59** |
|  |  | SYBR | TFRI | RV14-2 | 26.71 | 18.28 | 8.43 | 9.54 | -1.11 | 2.158456473 | **1.11** |  |  |  |  |  |
|  |  | SYBR | TFRI | RV14-3 | 27.23 | 18.97 | 8.26 | 9.54 | -1.28 | 2.428389769 | **1.28** |  |  |  |  |  |
|  |  | SYBR | TFRI | RV14-4 | 27.96 | 18.96 | 9 | 9.54 | -0.54 | 1.453972517 | **0.54** |  |  |  |  |  |
| RV 21 dpi |  | SYBR | TFRI | RV21-1 | 25.17 | 18.79 | 6.38 | 9.54 | -3.16 | 8.938297105 | **3.16** | -2.4575 | 5.492640999 | **2.4575** | 0.64 | **0.37** |
|  |  | SYBR | TFRI | RV21-2 | 25.72 | 18.66 | 7.06 | 9.54 | -2.48 | 5.578974665 | **2.48** |  |  |  |  |  |
|  |  | SYBR | TFRI | RV21-3 | 26.36 | 18.72 | 7.64 | 9.54 | -1.9 | 3.732131966 | **1.9** |  |  |  |  |  |
|  |  | SYBR | TFRI | RV21-4 | 26.13 | 18.88 | 7.25 | 9.54 | -2.29 | 4.890561111 | **2.29** |  |  |  |  |  |
| RV 28dpi |  | SYBR | TFRI | RV28-1 | 28.34 | 19.3 | 9.04 | 9.54 | -0.5 | 1.414213562 | **0.5** | -0.7275 | 1.655767378 | **0.7275** | 1.3 | **0.75** |
|  |  | SYBR | TFRI | RV28-2 | 28.21 | 19.43 | 8.78 | 9.54 | -0.76 | 1.693490625 | **0.76** |  |  |  |  |  |
|  |  | SYBR | TFRI | RV28-3 | 28.06 | 19.42 | 8.64 | 9.54 | -0.9 | 1.866065983 | **0.9** |  |  |  |  |  |
|  |  | SYBR | TFRI | RV28-4 | 28.53 | 19.74 | 8.79 | 9.54 | -0.75 | 1.681792831 | **0.75** |  |  |  |  |  |

| treatment | Fluor | Target | cDNA sample | AVERAGE CQ BY SAMPLE | AVERAGE GAPDH CQ BY SAMPLE | Normalize to GAPDH | Avg of controls | Normalized to control | Fold change in expresssion | LOG BASE 2 AVG EXPRESSION RESULTS FOR GRAPH | AVERAGE Normalized to CONTROL | FOLD CHANGE AVERAGE EXPRESSION | LOG BASE 2 AVG EXPRESSION RESULTS FOR GRAPH | StDev | SEM |
| --- | --- | --- | --- | --- | --- | --- | --- | --- | --- | --- | --- | --- | --- | --- | --- |
| SHAM CONTROL | SYBR | LCN2 | SHAM-1 | 24.93 | 19.63 | 5.3 | 6.3875 | -1.0875 | 2.125054733 | **1.0875** | 0 | 1 | **0** | 0.44 | **0.22** |
|  | SYBR | LCN2 | SHAM-2 | 26.22 | 19.77 | 6.45 | 6.3875 | 0.0625 | 0.9576032807 | **-0.0625** |  |  |  |  |  |
|  | SYBR | LCN2 | SHAM-3 | 25.87 | 19.3 | 6.57 | 6.3875 | 0.1825 | 0.8811747131 | **-0.1825** |  |  |  |  |  |
|  | SYBR | LCN2 | SHAM-4 | 26.53 | 19.3 | 7.23 | 6.3875 | 0.8425 | 0.5576763518 | **-0.8425** |  |  |  |  |  |
| RV 7 dpi | SYBR | LCN2 | RV7-1 | 24.85 | 19.07 | 5.78 | 6.3875 | -0.6075 | 1.523616694 | **0.6075** | -1.1225 | 2.177239326 | **1.1225** | 1.18 | **0.59** |
|  | SYBR | LCN2 | RV7-2 | 24.91 | 19.11 | 5.8 | 6.3875 | -0.5875 | 1.502640612 | **0.5875** |  |  |  |  |  |
|  | SYBR | LCN2 | RV7-3 | 23.69 | 18.9 | 4.79 | 6.3875 | -1.5975 | 3.026184609 | **1.5975** |  |  |  |  |  |
|  | SYBR | LCN2 | RV7-4 | 23.37 | 18.68 | 4.69 | 6.3875 | -1.6975 | 3.243384356 | **1.6975** |  |  |  |  |  |
| RV 14 dpi | SYBR | LCN2 | RV14-1 | 24.75 | 19.3 | 5.45 | 6.3875 | -0.9375 | 1.915206561 | **0.9375** | -1.1275 | 2.184798154 | **1.1275** | 1.18 | **0.59** |
|  | SYBR | LCN2 | RV14-2 | 23.72 | 18.28 | 5.44 | 6.3875 | -0.9475 | 1.928527877 | **0.9475** |  |  |  |  |  |
|  | SYBR | LCN2 | RV14-3 | 23.97 | 18.97 | 5 | 6.3875 | -1.3875 | 2.616249263 | **1.3875** |  |  |  |  |  |
|  | SYBR | LCN2 | RV14-4 | 24.11 | 18.96 | 5.15 | 6.3875 | -1.2375 | 2.357895858 | **1.2375** |  |  |  |  |  |
| RV 21 dpi | SYBR | LCN2 | RV21-1 | 23.36 | 18.79 | 4.57 | 6.3875 | -1.8175 | 3.524698852 | **1.8175** | -2.0975 | 4.279671316 | **2.0975** | 0.64 | **0.37** |
|  | SYBR | LCN2 | RV21-2 | 22.48 | 18.66 | 3.82 | 6.3875 | -2.5675 | 5.92781326 | **2.5675** |  |  |  |  |  |
|  | SYBR | LCN2 | RV21-3 | 23.25 | 18.72 | 4.53 | 6.3875 | -1.8575 | 3.623791625 | **1.8575** |  |  |  |  |  |
|  | SYBR | LCN2 | RV21-4 | 23.12 | 18.88 | 4.24 | 6.3875 | -2.1475 | 4.430593599 | **2.1475** |  |  |  |  |  |
| RV 28dpi | SYBR | LCN2 | RV28-1 | 24.13 | 19.3 | 4.83 | 6.3875 | -1.5575 | 2.943433431 | **1.5575** | -1.2625 | 2.399111152 | **1.2625** | 1.3 | **0.75** |
|  | SYBR | LCN2 | RV28-2 | 25.27 | 19.43 | 5.84 | 6.3875 | -0.5475 | 1.461550826 | **0.5475** |  |  |  |  |  |
|  | SYBR | LCN2 | RV28-3 | 25.48 | 19.42 | 6.06 | 6.3875 | -0.3275 | 1.254837023 | **0.3275** |  |  |  |  |  |
|  | SYBR | LCN2 | RV28-4 | 23.51 | 19.74 | 3.77 | 6.3875 | -2.6175 | 6.136857143 | **2.6175** |  |  |  |  |  |

| treatment | Fluor | Target | cDNA sample | AVERAGE CQ BY SAMPLE | AVERAGE GAPDH CQ BY SAMPLE | Normalize to GAPDH | Avg of controls | Normalized to control | Fold change in expresssion | LOG BASE 2 AVG EXPRESSION RESULTS FOR GRAPH | AVERAGE Normalized to CONTROL | FOLD CHANGE AVERAGE EXPRESSION | LOG BASE 2 AVG EXPRESSION RESULTS FOR GRAPH | StDev | SEM |
| --- | --- | --- | --- | --- | --- | --- | --- | --- | --- | --- | --- | --- | --- | --- | --- |
| SHAM CONTROL | SYBR | PTGS2 | SHAM-1 | 30.31 | 19.63 | 10.68 | 10.735 | -0.055 | 1.038859103 | **0.055** | 0 | 1 | **0** | 0.44 | **0.22** |
|  | SYBR | PTGS2 | SHAM-2 | 30.01 | 19.77 | 10.24 | 10.735 | -0.495 | 1.409320755 | **0.495** |  |  |  |  |  |
|  | SYBR | PTGS2 | SHAM-3 | 30.1 | 19.3 | 10.8 | 10.735 | 0.065 | 0.9559453176 | **-0.065** |  |  |  |  |  |
|  | SYBR | PTGS2 | SHAM-4 | 30.52 | 19.3 | 11.22 | 10.735 | 0.485 | 0.7144970699 | **-0.485** |  |  |  |  |  |
| RV 7 dpi | SYBR | PTGS2 | RV7-1 | 30.48 | 19.07 | 11.41 | 10.735 | 0.675 | 0.6263322193 | **-0.675** | 0.455 | 0.7295101721 | **-0.455** | 1.18 | **0.59** |
|  | SYBR | PTGS2 | RV7-2 | 30.02 | 19.11 | 10.91 | 10.735 | 0.175 | 0.8857675191 | **-0.175** |  |  |  |  |  |
|  | SYBR | PTGS2 | RV7-3 | 29.92 | 18.9 | 11.02 | 10.735 | 0.285 | 0.8207416088 | **-0.285** |  |  |  |  |  |
|  | SYBR | PTGS2 | RV7-4 | 30.1 | 18.68 | 11.42 | 10.735 | 0.685 | 0.6220058266 | **-0.685** |  |  |  |  |  |
| RV 14 dpi | SYBR | PTGS2 | RV14-1 | 30.64 | 19.3 | 11.34 | 10.735 | 0.605 | 0.6574713801 | **-0.605** | 0.8525 | 0.5538241998 | **-0.8525** | 1.18 | **0.59** |
|  | SYBR | PTGS2 | RV14-2 | 30.15 | 18.28 | 11.87 | 10.735 | 1.135 | 0.4553349168 | **-1.135** |  |  |  |  |  |
|  | SYBR | PTGS2 | RV14-3 | 30.6 | 18.97 | 11.63 | 10.735 | 0.895 | 0.5377471952 | **-0.895** |  |  |  |  |  |
|  | SYBR | PTGS2 | RV14-4 | 30.47 | 18.96 | 11.51 | 10.735 | 0.775 | 0.5843886243 | **-0.775** |  |  |  |  |  |
| RV 21 dpi | SYBR | PTGS2 | RV21-1 | 29.83 | 18.79 | 11.04 | 10.735 | 0.305 | 0.8094422165 | **-0.305** | 0.5375 | 0.6889637585 | **-0.5375** | 0.64 | **0.37** |
|  | SYBR | PTGS2 | RV21-2 | 30.16 | 18.66 | 11.5 | 10.735 | 0.765 | 0.5884533686 | **-0.765** |  |  |  |  |  |
|  | SYBR | PTGS2 | RV21-3 | 30.66 | 18.72 | 11.94 | 10.735 | 1.205 | 0.4337693436 | **-1.205** |  |  |  |  |  |
|  | SYBR | PTGS2 | RV21-4 | 29.49 | 18.88 | 10.61 | 10.735 | -0.125 | 1.090507733 | **0.125** |  |  |  |  |  |
| RV 28dpi | SYBR | PTGS2 | RV28-1 | 30.83 | 19.3 | 11.53 | 10.735 | 0.795 | 0.5763431734 | **-0.795** | 0.46 | 0.7269862587 | **-0.46** | 1.3 | **0.75** |
|  | SYBR | PTGS2 | RV28-2 | 30.78 | 19.43 | 11.35 | 10.735 | 0.615 | 0.6529298935 | **-0.615** |  |  |  |  |  |
|  | SYBR | PTGS2 | RV28-3 | 30.18 | 19.42 | 10.76 | 10.735 | 0.025 | 0.9828205985 | **-0.025** |  |  |  |  |  |
|  | SYBR | PTGS2 | RV28-4 | 30.88 | 19.74 | 11.14 | 10.735 | 0.405 | 0.7552362928 | **-0.405** |  |  |  |  |  |

| treatment | Fluor | Target | cDNA sample | AVERAGE CQ BY SAMPLE | AVERAGE GAPDH CQ BY SAMPLE | Normalize to GAPDH | Avg of controls | Normalized to control | Fold change in expresssion | LOG BASE 2 AVG EXPRESSION RESULTS FOR GRAPH | AVERAGE Normalized to CONTROL | FOLD CHANGE AVERAGE EXPRESSION | LOG BASE 2 AVG EXPRESSION RESULTS FOR GRAPH | StDev | SEM |
| --- | --- | --- | --- | --- | --- | --- | --- | --- | --- | --- | --- | --- | --- | --- | --- |
| SHAM CONTROL | SYBR | HO-1 | SHAM-1 | 25.24 | 19.63 | 5.61 | 5.835 | -0.225 | 1.168777249 | **0.225** | 0 | 1 | **0** | 0.44 | **0.22** |
|  | SYBR | HO-1 | SHAM-2 | 25.94 | 19.77 | 6.17 | 5.835 | 0.335 | 0.7927841366 | **-0.335** |  |  |  |  |  |
|  | SYBR | HO-1 | SHAM-3 | 24.95 | 19.3 | 5.65 | 5.835 | -0.185 | 1.136816973 | **0.185** |  |  |  |  |  |
|  | SYBR | HO-1 | SHAM-4 | 25.21 | 19.3 | 5.91 | 5.835 | 0.075 | 0.949342121 | **-0.075** |  |  |  |  |  |
| RV 7 dpi | SYBR | HO-1 | RV7-1 | 25.25 | 19.07 | 6.18 | 5.835 | 0.345 | 0.7873079766 | **-0.345** | 0.1 | 0.9330329915 | **-0.1** | 1.18 | **0.59** |
|  | SYBR | HO-1 | RV7-2 | 24.61 | 19.11 | 5.5 | 5.835 | -0.335 | 1.261377409 | **0.335** |  |  |  |  |  |
|  | SYBR | HO-1 | RV7-3 | 24.64 | 18.9 | 5.74 | 5.835 | -0.095 | 1.068065408 | **0.095** |  |  |  |  |  |
|  | SYBR | HO-1 | RV7-4 | 25 | 18.68 | 6.32 | 5.835 | 0.485 | 0.7144970699 | **-0.485** |  |  |  |  |  |
| RV 14 dpi | SYBR | HO-1 | RV14-1 | 24.39 | 19.3 | 5.09 | 5.835 | -0.745 | 1.675974269 | **0.745** | -0.465 | 1.380317353 | **0.465** | 1.18 | **0.59** |
|  | SYBR | HO-1 | RV14-2 | 23.58 | 18.28 | 5.3 | 5.835 | -0.535 | 1.448942155 | **0.535** |  |  |  |  |  |
|  | SYBR | HO-1 | RV14-3 | 24.3 | 18.97 | 5.33 | 5.835 | -0.505 | 1.419123356 | **0.505** |  |  |  |  |  |
|  | SYBR | HO-1 | RV14-4 | 24.72 | 18.96 | 5.76 | 5.835 | -0.075 | 1.053361036 | **0.075** |  |  |  |  |  |
| RV 21 dpi | SYBR | HO-1 | RV21-1 | 23.17 | 18.79 | 4.38 | 5.835 | -1.455 | 2.74156561 | **1.455** | -1.265 | 2.403272099 | **1.265** | 0.64 | **0.37** |
|  | SYBR | HO-1 | RV21-2 | 23.31 | 18.66 | 4.65 | 5.835 | -1.185 | 2.273633946 | **1.185** |  |  |  |  |  |
|  | SYBR | HO-1 | RV21-3 | 23.54 | 18.72 | 4.82 | 5.835 | -1.015 | 2.020902893 | **1.015** |  |  |  |  |  |
|  | SYBR | HO-1 | RV21-4 | 23.31 | 18.88 | 4.43 | 5.835 | -1.405 | 2.648177821 | **1.405** |  |  |  |  |  |
| RV 28dpi | SYBR | HO-1 | RV28-1 | 24.87 | 19.3 | 5.57 | 5.835 | -0.265 | 1.20163605 | **0.265** | -0.2625 | 1.199555576 | **0.2625** | 1.3 | **0.75** |
|  | SYBR | HO-1 | RV28-2 | 25.06 | 19.43 | 5.63 | 5.835 | -0.205 | 1.152686347 | **0.205** |  |  |  |  |  |
|  | SYBR | HO-1 | RV28-3 | 25.07 | 19.42 | 5.65 | 5.835 | -0.185 | 1.136816973 | **0.185** |  |  |  |  |  |
|  | SYBR | HO-1 | RV28-4 | 25.18 | 19.74 | 5.44 | 5.835 | -0.395 | 1.31494276 | **0.395** |  |  |  |  |  |

| treatment | Fluor | Target | cDNA sample | AVERAGE CQ BY SAMPLE | AVERAGE GAPDH CQ BY SAMPLE | Normalize to GAPDH | Avg of controls | Normalized to control | Fold change in expresssion | LOG BASE 2 AVG EXPRESSION RESULTS FOR GRAPH | AVERAGE Normalized to CONTROL | FOLD CHANGE AVERAGE EXPRESSION | LOG BASE 2 AVG EXPRESSION RESULTS FOR GRAPH | StDev | SEM |
| --- | --- | --- | --- | --- | --- | --- | --- | --- | --- | --- | --- | --- | --- | --- | --- |
| SHAM CONTROL | SYBR | GPX4 | SHAM-1 | 29.43 | 19.63 | 9.8 | 10.4075 | -0.6075 | 1.523616694 | **0.6075** | 0 | 1 | **0** | 0.44 | **0.22** |
|  | SYBR | GPX4 | SHAM-2 | 29.78 | 19.77 | 10.01 | 10.4075 | -0.3975 | 1.317223358 | **0.3975** |  |  |  |  |  |
|  | SYBR | GPX4 | SHAM-3 | 29.12 | 19.3 | 9.82 | 10.4075 | -0.5875 | 1.502640612 | **0.5875** |  |  |  |  |  |
|  | SYBR | GPX4 | SHAM-4 | 31.3 | 19.3 | 12 | 10.4075 | 1.5925 | 0.3315963427 | **-1.5925** |  |  |  |  |  |
| RV 7 dpi | SYBR | GPX4 | RV7-1 | 30.14 | 19.07 | 11.07 | 10.4075 | 0.6625 | 0.6317825521 | **-0.6625** | 0.215 | 0.8615461597 | **-0.215** | 1.18 | **0.59** |
|  | SYBR | GPX4 | RV7-2 | 28.53 | 19.11 | 9.42 | 10.4075 | -0.9875 | 1.982746175 | **0.9875** |  |  |  |  |  |
|  | SYBR | GPX4 | RV7-3 | 29.64 | 18.9 | 10.74 | 10.4075 | 0.3325 | 0.7941591178 | **-0.3325** |  |  |  |  |  |
|  | SYBR | GPX4 | RV7-4 | 29.94 | 18.68 | 11.26 | 10.4075 | 0.8525 | 0.5538241998 | **-0.8525** |  |  |  |  |  |
| RV 14 dpi | SYBR | GPX4 | RV14-1 | 30.77 | 19.3 | 11.47 | 10.4075 | 1.0625 | 0.4788016403 | **-1.0625** | 1.325 | 0.3991491932 | **-1.325** | 1.18 | **0.59** |
|  | SYBR | GPX4 | RV14-2 | 29.87 | 18.28 | 11.59 | 10.4075 | 1.1825 | 0.4405873565 | **-1.1825** |  |  |  |  |  |
|  | SYBR | GPX4 | RV14-3 | 30.06 | 18.97 | 11.09 | 10.4075 | 0.6825 | 0.623084615 | **-0.6825** |  |  |  |  |  |
|  | SYBR | GPX4 | RV14-4 | 31.74 | 18.96 | 12.78 | 10.4075 | 2.3725 | 0.1931106987 | **-2.3725** |  |  |  |  |  |
| RV 21 dpi | SYBR | GPX4 | RV21-1 | 32.05 | 18.79 | 13.26 | 10.4075 | 2.8525 | 0.13845605 | **-2.8525** | 2.3275 | 0.1992290596 | **-2.3275** | 0.64 | **0.37** |
|  | SYBR | GPX4 | RV21-2 | 32.16 | 18.66 | 13.5 | 10.4075 | 3.0925 | 0.1172370113 | **-3.0925** |  |  |  |  |  |
|  | SYBR | GPX4 | RV21-3 | 28.88 | 18.72 | 10.16 | 10.4075 | -0.2475 | 1.187148161 | **0.2475** |  |  |  |  |  |
|  | SYBR | GPX4 | RV21-4 | 32.9 | 18.88 | 14.02 | 10.4075 | 3.6125 | 0.08175778946 | **-3.6125** |  |  |  |  |  |
| RV 28dpi | SYBR | GPX4 | RV28-1 | 29.11 | 19.3 | 9.81 | 10.4075 | -0.5975 | 1.513092304 | **0.5975** | 1.0475 | 0.4838058101 | **-1.0475** | 1.3 | **0.75** |
|  | SYBR | GPX4 | RV28-2 | 30.07 | 19.43 | 10.64 | 10.4075 | 0.2325 | 0.8511586675 | **-0.2325** |  |  |  |  |  |
|  | SYBR | GPX4 | RV28-3 | 32.05 | 19.42 | 12.63 | 10.4075 | 2.2225 | 0.2142697361 | **-2.2225** |  |  |  |  |  |
|  | SYBR | GPX4 | RV28-4 | 32.48 | 19.74 | 12.74 | 10.4075 | 2.3325 | 0.1985397795 | **-2.3325** |  |  |  |  |  |

| treatment | Fluor | Target | cDNA sample | AVERAGE CQ BY SAMPLE | AVERAGE GAPDH CQ BY SAMPLE | Normalize to GAPDH | Avg of controls | Normalized to control | Fold change in expresssion | LOG BASE 2 AVG EXPRESSION RESULTS FOR GRAPH | AVERAGE Normalized to CONTROL | FOLD CHANGE AVERAGE EXPRESSION | LOG BASE 2 AVG EXPRESSION RESULTS FOR GRAPH | StDev | SEM |
| --- | --- | --- | --- | --- | --- | --- | --- | --- | --- | --- | --- | --- | --- | --- | --- |
| SHAM CONTROL | SYBR | ITGAM | SHAM-1 | 33.88 | 19.63 | 14.25 | 14.955 | -0.705 | 1.630144665 | **0.705** | 0 | 1 | **0** | 0.44 | **0.22** |
|  | SYBR | ITGAM | SHAM-2 | 35.25 | 19.77 | 15.48 | 14.955 | 0.525 | 0.6949591099 | **-0.525** |  |  |  |  |  |
|  | SYBR | ITGAM | SHAM-3 | 34.18 | 19.3 | 14.88 | 14.955 | -0.075 | 1.053361036 | **0.075** |  |  |  |  |  |
|  | SYBR | ITGAM | SHAM-4 | 34.51 | 19.3 | 15.21 | 14.955 | 0.255 | 0.8379871347 | **-0.255** |  |  |  |  |  |
| RV 7 dpi | SYBR | ITGAM | RV7-1 | 31.75 | 19.07 | 12.68 | 14.955 | -2.275 | 4.839976357 | **2.275** | -2.5275 | 5.765716894 | **2.5275** | 1.18 | **0.59** |
|  | SYBR | ITGAM | RV7-2 | 31.13 | 19.11 | 12.02 | 14.955 | -2.935 | 7.647562541 | **2.935** |  |  |  |  |  |
|  | SYBR | ITGAM | RV7-3 | 30.99 | 18.9 | 12.09 | 14.955 | -2.865 | 7.285358669 | **2.865** |  |  |  |  |  |
|  | SYBR | ITGAM | RV7-4 | 31.6 | 18.68 | 12.92 | 14.955 | -2.035 | 4.098227292 | **2.035** |  |  |  |  |  |
| RV 14 dpi | SYBR | ITGAM | RV14-1 | 31.85 | 19.3 | 12.55 | 14.955 | -2.405 | 5.296355642 | **2.405** | -2.39 | 5.241573615 | **2.39** | 1.18 | **0.59** |
|  | SYBR | ITGAM | RV14-2 | 30.61 | 18.28 | 12.33 | 14.955 | -2.625 | 6.168843302 | **2.625** |  |  |  |  |  |
|  | SYBR | ITGAM | RV14-3 | 31.36 | 18.97 | 12.39 | 14.955 | -2.565 | 5.917550037 | **2.565** |  |  |  |  |  |
|  | SYBR | ITGAM | RV14-4 | 31.95 | 18.96 | 12.99 | 14.955 | -1.965 | 3.904127043 | **1.965** |  |  |  |  |  |
| RV 21 dpi | SYBR | ITGAM | RV21-1 | 35.69 | 18.79 | 16.9 | 14.955 | 1.945 | 0.2597147758 | **-1.945** | 1.73 | 0.3014519569 | **-1.73** | 0.64 | **0.37** |
|  | SYBR | ITGAM | RV21-2 | 36.63 | 18.66 | 17.97 | 14.955 | 3.015 | 0.1237070821 | **-3.015** |  |  |  |  |  |
|  | SYBR | ITGAM | RV21-3 | 35.5 | 18.72 | 16.78 | 14.955 | 1.825 | 0.2822411012 | **-1.825** |  |  |  |  |  |
|  | SYBR | ITGAM | RV21-4 | 33.97 | 18.88 | 15.09 | 14.955 | 0.135 | 0.9106698336 | **-0.135** |  |  |  |  |  |
| RV 28dpi | SYBR | ITGAM | RV28-1 | 36.5 | 19.3 | 17.2 | 14.955 | 2.245 | 0.210953949 | **-2.245** | 1.795 | 0.2881715867 | **-1.795** | 1.3 | **0.75** |
|  | SYBR | ITGAM | RV28-2 | 36.11 | 19.43 | 16.68 | 14.955 | 1.725 | 0.3024985223 | **-1.725** |  |  |  |  |  |
|  | SYBR | ITGAM | RV28-3 | 35.65 | 19.42 | 16.23 | 14.955 | 1.275 | 0.4132251591 | **-1.275** |  |  |  |  |  |
|  | SYBR | ITGAM | RV28-4 | 36.63 | 19.74 | 16.89 | 14.955 | 1.935 | 0.2615212349 | **-1.935** |  |  |  |  |  |

| treatment | Fluor | Target | cDNA sample | AVERAGE CQ BY SAMPLE | AVERAGE GAPDH CQ BY SAMPLE | Normalize to GAPDH | Avg of controls | Normalized to control | Fold change in expression | LOG BASE 2 AVG EXPRESSION RESULTS FOR GRAPH | AVERAGE Normalized to CONTROL | FOLD CHANGE AVERAGE EXPRESSION | LOG BASE 2 AVG EXPRESSION RESULTS FOR GRAPH | StDev | SEM |
| --- | --- | --- | --- | --- | --- | --- | --- | --- | --- | --- | --- | --- | --- | --- | --- |
| SHAM CONTROL | SYBR | NRF2 | SHAM-1 | 30.4 | 19.63 | 10.77 | 10.855 | -0.085 | 1.060687741 | **0.085** | 0 | 1 | **0** | 0.44 | **0.22** |
|  | SYBR | NRF2 | SHAM-2 | 30.79 | 19.77 | 11.02 | 10.855 | 0.165 | 0.8919285194 | **-0.165** |  |  |  |  |  |
|  | SYBR | NRF2 | SHAM-3 | 30.01 | 19.3 | 10.71 | 10.855 | -0.145 | 1.105730653 | **0.145** |  |  |  |  |  |
|  | SYBR | NRF2 | SHAM-4 | 30.22 | 19.3 | 10.92 | 10.855 | 0.065 | 0.9559453176 | **-0.065** |  |  |  |  |  |
| RV 7 dpi | SYBR | NRF2 | RV7-1 | 30.59 | 19.07 | 11.52 | 10.855 | 0.665 | 0.6306887044 | **-0.665** | 0.385 | 0.7657789985 | **-0.385** | 1.18 | **0.59** |
|  | SYBR | NRF2 | RV7-2 | 29.54 | 19.11 | 10.43 | 10.855 | -0.425 | 1.342572503 | **0.425** |  |  |  |  |  |
|  | SYBR | NRF2 | RV7-3 | 30.21 | 18.9 | 11.31 | 10.855 | 0.455 | 0.7295101721 | **-0.455** |  |  |  |  |  |
|  | SYBR | NRF2 | RV7-4 | 30.38 | 18.68 | 11.7 | 10.855 | 0.845 | 0.5567108091 | **-0.845** |  |  |  |  |  |
| RV 14 dpi | SYBR | NRF2 | RV14-1 | 31.03 | 19.3 | 11.73 | 10.855 | 0.875 | 0.5452538663 | **-0.875** | 0.64 | 0.6417129488 | **-0.64** | 1.18 | **0.59** |
|  | SYBR | NRF2 | RV14-2 | 29.99 | 18.28 | 11.71 | 10.855 | 0.855 | 0.5528653267 | **-0.855** |  |  |  |  |  |
|  | SYBR | NRF2 | RV14-3 | 30.25 | 18.97 | 11.28 | 10.855 | 0.425 | 0.7448387316 | **-0.425** |  |  |  |  |  |
|  | SYBR | NRF2 | RV14-4 | 30.22 | 18.96 | 11.26 | 10.855 | 0.405 | 0.7552362928 | **-0.405** |  |  |  |  |  |
| RV 21 dpi | SYBR | NRF2 | RV21-1 | 30.16 | 18.79 | 11.37 | 10.855 | 0.515 | 0.6997929328 | **-0.515** | 0.62 | 0.6506709277 | **-0.62** | 0.64 | **0.37** |
|  | SYBR | NRF2 | RV21-2 | 30.19 | 18.66 | 11.53 | 10.855 | 0.675 | 0.6263322193 | **-0.675** |  |  |  |  |  |
|  | SYBR | NRF2 | RV21-3 | 30.41 | 18.72 | 11.69 | 10.855 | 0.835 | 0.560583039 | **-0.835** |  |  |  |  |  |
|  | SYBR | NRF2 | RV21-4 | 30.19 | 18.88 | 11.31 | 10.855 | 0.455 | 0.7295101721 | **-0.455** |  |  |  |  |  |
| RV 28dpi | SYBR | NRF2 | RV28-1 | 31.08 | 19.3 | 11.78 | 10.855 | 0.925 | 0.526680518 | **-0.925** | 0.3925 | 0.7618083468 | **-0.3925** | 1.3 | **0.75** |
|  | SYBR | NRF2 | RV28-2 | 30.64 | 19.43 | 11.21 | 10.855 | 0.355 | 0.7818696431 | **-0.355** |  |  |  |  |  |
|  | SYBR | NRF2 | RV28-3 | 30.71 | 19.42 | 11.29 | 10.855 | 0.435 | 0.7396937546 | **-0.435** |  |  |  |  |  |
|  | SYBR | NRF2 | RV28-4 | 30.45 | 19.74 | 10.71 | 10.855 | -0.145 | 1.105730653 | **0.145** |  |  |  |  |  |

| treatment | Fluor | Target | cDNA sample | AVERAGE CQ BY SAMPLE | AVERAGE GAPDH CQ BY SAMPLE | Normalize to GAPDH | Avg of controls | Normalized to control | Fold change in expression | LOG BASE 2 AVG EXPRESSION RESULTS FOR GRAPH | AVERAGE Normalized to CONTROL | FOLD CHANGE AVERAGE EXPRESSION | LOG BASE 2 AVG EXPRESSION RESULTS FOR GRAPH | StDev | SEM |
| --- | --- | --- | --- | --- | --- | --- | --- | --- | --- | --- | --- | --- | --- | --- | --- |
| SHAM CONTROL | SYBR | SLC7A11 | SHAM-1 | 29.79 | 19.63 | 10.16 | 10.39 | -0.23 | 1.172834949 | **0.23** | 0 | 1 | **0** | 0.44 | **0.22** |
|  | SYBR | SLC7A11 | SHAM-2 | 30.04 | 19.77 | 10.27 | 10.39 | -0.12 | 1.086734863 | **0.12** |  |  |  |  |  |
|  | SYBR | SLC7A11 | SHAM-3 | 29.78 | 19.3 | 10.48 | 10.39 | 0.09 | 0.9395227492 | **-0.09** |  |  |  |  |  |
|  | SYBR | SLC7A11 | SHAM-4 | 29.95 | 19.3 | 10.65 | 10.39 | 0.26 | 0.8350879194 | **-0.26** |  |  |  |  |  |
| RV 7 dpi | SYBR | SLC7A11 | RV7-1 | 30.01 | 19.07 | 10.94 | 10.39 | 0.55 | 0.6830201284 | **-0.55** | 0.605 | 0.6574713801 | **-0.605** | 1.18 | **0.59** |
|  | SYBR | SLC7A11 | RV7-2 | 29.94 | 19.11 | 10.83 | 10.39 | 0.44 | 0.7371346086 | **-0.44** |  |  |  |  |  |
|  | SYBR | SLC7A11 | RV7-3 | 29.69 | 18.9 | 10.79 | 10.39 | 0.4 | 0.7578582833 | **-0.4** |  |  |  |  |  |
|  | SYBR | SLC7A11 | RV7-4 | 30.1 | 18.68 | 11.42 | 10.39 | 1.03 | 0.4897101488 | **-1.03** |  |  |  |  |  |
| RV 14 dpi | SYBR | SLC7A11 | RV14-1 | 30.13 | 19.3 | 10.83 | 10.39 | 0.44 | 0.7371346086 | **-0.44** | 1.0225 | 0.4922625867 | **-1.0225** | 1.18 | **0.59** |
|  | SYBR | SLC7A11 | RV14-2 | 30.1 | 18.28 | 11.82 | 10.39 | 1.43 | 0.3711308927 | **-1.43** |  |  |  |  |  |
|  | SYBR | SLC7A11 | RV14-3 | 29.89 | 18.97 | 10.92 | 10.39 | 0.53 | 0.6925547341 | **-0.53** |  |  |  |  |  |
|  | SYBR | SLC7A11 | RV14-4 | 31.04 | 18.96 | 12.08 | 10.39 | 1.69 | 0.309926925 | **-1.69** |  |  |  |  |  |
| RV 21 dpi | SYBR | SLC7A11 | RV21-1 | 30.26 | 18.79 | 11.47 | 10.39 | 1.08 | 0.4730288234 | **-1.08** | 1.0575 | 0.4804639192 | **-1.0575** | 0.64 | **0.37** |
|  | SYBR | SLC7A11 | RV21-2 | 30.24 | 18.66 | 11.58 | 10.39 | 1.19 | 0.4383028607 | **-1.19** |  |  |  |  |  |
|  | SYBR | SLC7A11 | RV21-3 | 30.29 | 18.72 | 11.57 | 10.39 | 1.18 | 0.4413514981 | **-1.18** |  |  |  |  |  |
|  | SYBR | SLC7A11 | RV21-4 | 30.05 | 18.88 | 11.17 | 10.39 | 0.78 | 0.5823667932 | **-0.78** |  |  |  |  |  |
| RV 28dpi | SYBR | SLC7A11 | RV28-1 | 30.12 | 19.3 | 10.82 | 10.39 | 0.43 | 0.7422617853 | **-0.43** | 0.3825 | 0.7671071428 | **-0.3825** | 1.3 | **0.75** |
|  | SYBR | SLC7A11 | RV28-2 | 30.06 | 19.43 | 10.63 | 10.39 | 0.24 | 0.8467453124 | **-0.24** |  |  |  |  |  |
|  | SYBR | SLC7A11 | RV28-3 | 30.52 | 19.42 | 11.1 | 10.39 | 0.71 | 0.6113201388 | **-0.71** |  |  |  |  |  |
|  | SYBR | SLC7A11 | RV28-4 | 30.28 | 19.74 | 10.54 | 10.39 | 0.15 | 0.9012504626 | **-0.15** |  |  |  |  |  |

Data processed from raw data from Bio-Rad qPCR machine 6.85 Gy

IL-1beta

| Sham | 7 | 14 | 21 |
| --- | --- | --- | --- |
| 0.61 | 0.22 | 1.46 | 3.39 |
| 1.58 | 0.40 | 2.00 | 4.47 |
| 1.037 | 0.15 | 0.92 | 3.86 |
| 0.98 | 1.33 | 1.72 | 3.06 |
| 0.99 | 1.62 | 1.37 | 3.06 |
| 1.03 | 1.86 | 1.74 | 3.02 |

SAA

| sham | 7 | 14 | 21 |
| --- | --- | --- | --- |
| 1.1 | 0.44 | 3.01 | 4.11 |
| 1.11 | 0.58 | 2.37 | 2.03 |
| 0.82 | 0.49 | 1.3 | 2.46 |
| 1.11 | 0.55 | 3.51 | 1.96 |
| 0.73 | 0.96 | 3.88 | 2.96 |
| 1.23 | 0.64 | 3.64 | 2.72 |

Iron assay data

7.9 Gy study

7.9 Gy data

Western blot data

7.9 Gy

|  | ferritin | b-actin | norm |
| --- | --- | --- | --- |
| S1 | 5350 | 39300 | 0.136132 |
| s2 | 10700 | 47000 | 0.22766 |
| s3 | 6840 | 64600 | 0.105882 |
| s4 | 11600 | 66500 | 0.174436 |
| 5..1 | 12500 | 42400 | 0.294811 |
| 5..2 | 30900 | 48000 | 0.64375 |
| 5..3 | 786 | 36100 | 0.021773 |
| 5..4 | 7540 | 73100 | 0.103146 |
| 12..1 | 15600 | 40700 | 0.383292 |
| 12..2 | 21800 | 41400 | 0.52657 |
| 12..3 | 18200 | 63700 | 0.285714 |
| 12..4 | 27000 | 91000 | 0.296703 |
| 16-1 | 42100 | 53600 | 0.785448 |
| 16-2 | 53000 | 49900 | 1.062124 |
| 16-3 | 28900 | 99600 | 0.290161 |
| 16-4 | 29000 | 66300 | 0.437406 |
| 27-1 | 27300 | 34300 | 0.795918 |
| 27-2 | 64600 | 56400 | 1.14539 |
| 27-3 | 25700 | 13500 | 1.903704 |

6.85 Gy
