# Supplementary material for: Cell death and iron deposition in the liver in two murine models of acute radiation syndrome
Source: PLoS One. 2025 May 29;20(5):e0324361. doi: 10.1371/journal.pone.0324361 (PMC12121821; doi:10.1371/journal.pone.0324361)
Supplement: Attachment — Submitted filename: Response to reviewers PLoS One.docx [file pone.0324361.s005.docx]

April 4, 2025

PONE-D-24-37950

Cell Death and Iron Deposition in the Liver in two Murine Models of Acute Radiation Syndrome

We thank the Editor and Reviewers for the careful analysis of our manuscript. We have responded to all questions (see below), and provide a revised version of our manuscript.

Best Regards,

Regina Day

Journal Requirements:

1. Please ensure that your manuscript meets PLOS ONE's style requirements, including those for file naming.

We have double checked the style requirements, including file names, and have made the required corrections.

2. Thank you for your submission to PLOS ONE. We note that your study design may include death of a regulated animal as a likely outcome or planned experimental endpoint. At this time, we request that you please report additional details in your Methods section regarding animal care and use for the survival study, as per our editorial guidelines (http://journals.plos.org/plosone/s/submission-guidelines#loc-humane-endpoints). For easy reference, we have attached a checklist that may be relevant for your submission. Please complete all items on the checklist at the following link. Please upload the completed checklist as file type “Other” when resubmitting your manuscript. This document is for internal journal use only and will not be published if your article is accepted. We very much appreciate your attention to these requests and support of improved reporting standards in PLOS ONE submissions.

The Checklist is now uploaded as file type “Other” and includes responses to all ARRIVE criteria.

3. We note that the grant information you provided in the ‘Funding Information’ and ‘Financial Disclosure’ sections do not match. When you resubmit, please ensure that you provide the correct grant numbers for the awards you received for your study in the ‘Funding Information’ section.

We have double checked our Funding Information for correct grant numbers for the supporting awards.

4. We note that your Data Availability Statement is currently as follows:

“All relevant data are within the manuscript and its Supporting Information files.”

We now include additional Supporting Information Files that include all raw data from western blots, iron assays, and qPCR experiments. The file containing all raw data is “

5. PLOS ONE now requires that authors provide the original uncropped and unadjusted images underlying all blot or gel results reported in a submission’s figures or Supporting Information files.

In your cover letter, please note whether your blot/gel image data are in Supporting Information or posted at a public data repository, provide the repository URL if relevant, and provide specific details as to which raw blot/gel images, if any, are not available. Email us at plosone@plos.org if you have any questions.

We include all blot/gel images in the file “S1_raw_images.pdf” as instructed.

6. We note that you have included the phrase “data not shown” in your manuscript. Unfortunately, this does not meet our data sharing requirements. PLOS does not permit references to inaccessible data. We require that authors provide all relevant data within the paper, Supporting Information files, or in an acceptable, public repository. Please add a citation to support this phrase or upload the data that corresponds with these findings to a stable repository (such as Figshare or Dryad) and provide and URLs, DOIs, or accession numbers that may be used to access these data. Or, if the data are not a core part of the research being presented in your study, we ask that you remove the phrase that refers to these data.

We have removed references to data that is not shown.

7. Your ethics statement should only appear in the Methods section of your manuscript. If your ethics statement is written in any section besides the Methods, please move it to the Methods section and delete it from any other section. Please ensure that your ethics statement is included in your manuscript, as the ethics statement entered into the online submission form will not be published alongside your manuscript.

The Ethics Statement is now included in the Methods.

8. Please review your reference list to ensure that it is complete and correct. If you have cited papers that have been retracted, please include the rationale for doing so in the manuscript text, or remove these references and replace them with relevant current references. Any changes to the reference list should be mentioned in the rebuttal letter that accompanies your revised manuscript. If you need to cite a retracted article, indicate the article’s retracted status in the References list and also include a citation and full reference for the retraction notice.

We have searched our list of references for articles that had been retracted or modified. To the best of our knowledge, and based on a PubMed search of each article, the publications we cited are correct, and none have been retracted.

9) Thank you for stating the following financial disclosure:

"Defense Medical Research and Materiel Command, Radiation Health Effects Research Program, Joint Program Committee 7, grant numbers DM178018 and 1 I80 VP000264-01"

Please state what role the funders took in the study. If the funders had no role, please state: "The funders had no role in study design, data collection and analysis, decision to publish, or preparation of the manuscript." If this statement is not correct you must amend it as needed.

The funders had no role in the study design, data collection and analysis, decision to publish, or preparation of the manuscript. This amended Role of Funder statement is included in the cover letter. We note that PLoS One will change the online submission form on our behalf.

10) Please note that funding information should not appear in the Acknowledgments section or other areas of your manuscript. We will only publish funding information present in the Funding Statement section of the online submission form. Please remove any funding-related text from the manuscript.

We have removed the funding statement from the Acknowledgements.

Additional Editor Comments:

While both reviewers find the study potentially interesting, they have expressed significant concerns regarding the lack of clear evidence demonstrating apoptotic cells in the liver. Therefore, the authors should address this issue.

We have edited the manuscript to provide additional data as well as restatement of our conclusions as recommended by the Reviewers. A point-by-point response is provided below.

Specific Reviewer comments:

Reviewer #1: In this manuscript, the authors investigated the relationship between tissue injury and iron-related proteins in mouse liver with high-dose total body irradiation (TBI). They already demonstrated 7.9 Gy TBI induced tissue injury but not 6.85 Gy. TBI was reported to induce the hemolysis leading to the release of iron, and they confirmed the effects of released iron on TBI-induced tissue injury. At first, they might intend to connect ferroptosis with tissue injury. However non-toxic 6.85 Gy treatment showed the clear changes related to ferroptosis, but toxic 7.9 Gy treatment did not.

Our investigation of ferroptosis indicates that there are markers of ferroptosis present in the liver following 6.85 Gy but not in 7.9 Gy. Conclusive evidence of ferroptosis should include either data showing oxidized lipid modification of proteins or direct lipid oxidation. We were unable to obtain this conclusive evidence with several assays that we conducted. We now state in the Results of the 7.9 Gy study that our data suggest that markers of ferroptosis are not present; we also state in the Results of the 6.85 Gy section that markers of ferroptosis are present. We now state in the Conclusions section that the changes in ferroptosis markers are present following 6.85 Gy exposure, but that additional data would be required to provide proof of ferroptosis.

Although there is some difference related to iron transport between two conditions, ferroptosis was not the major reason for tissue injury. In contrast, they described “7.9 Gy induced liver caspase-3 activation consistent with apoptosis. In contrast, 6.85 Gy induced markers of ferroptosis but not of apoptosis” in abstract and “Caspase-3 activation was not observed at this lower dose of radiation (data not shown)” in discussion. These parts imply the importance of apoptosis or caspase activation in liver tissue injury, but the authors did not show the results examining caspase-3 activity in 6.85 Gy-treated sample.

We examined Caspase-3 activation after 6.85 Gy exposure, and our data was negative. This negative data is now shown in Figure S1.

Reviewer #2: In this study, Day and colleagues analyzed the kinetics of proteins and genes in the liver of mice subjected to total body irradiation (TBI). They found that TBI may induce iron accumulation, as indicated by increased levels of ferritin protein. Additionally, upregulation of HO-1 may be associated with increased oxidative stress in the liver.

This study provides valuable histopathological data that could help evaluate the degree of post-irradiation liver injury not only in mice but also in humans. The introduction is well-written and explains the mechanisms underlying TBI-induced iron dysregulation. However, some statements lack adequate scientific support, and cell death was not extensively analyzed in the data provided. Overall, while the value of this work is undeniable, this reviewer recommends that the authors carefully revise the manuscript as outlined below before publication in PLOS ONE.

Major Comments

1. Although the authors state that TBI induced hepatocellular apoptosis, Figure 3 does not show a clear upregulation of activated caspase-3 over the course of the experiment. To support this claim, apoptosis induction should also be confirmed by immunohistochemical analysis of the liver. The number of activated caspase-positive hepatocytes should be quantified for statistical analysis.

We were only able to show caspase-3 activation transiently at 5 days post-irradiation. In the future, a tighter time course, with more early time points, may be able to provide better proof of activation of markers of apoptosis in the liver. We now provide IHC

2. The title of this paper is somewhat misleading, as markers of ferroptosis were not significantly upregulated in the liver of TBI mice. It is generally recommended that the authors demonstrate inhibition of cell death using ferroptosis inhibitors to substantiate claims of ferroptosis involvement.

A number of studies have demonstrated that the inclusion of ferroptosis inhibitors or iron chelators reduce tissue pathology following total body or partial body irradiation. Unfortunately, we do not have specific funding to support these animal studies at this time. Without a specific peer-reviewed grant to perform this work, we are unable to perform these studies. We hope to obtain funding to pursue this work in the future. We have amended the title to include iron deposition and we have removed cell death and ferroptosis.

Minor Comments

1. The authors state that TBI induces erythrophagocytosis by Kupffer cells (KCs) in the liver (e.g., Figures 1 and 6). To support this claim, macrophages should be specified using CD68 or F4/80 staining.

These cells were identified by a certified veterinary pathologist. We have removed this claim from the manuscript.

2. The authors claim that the Prussian Blue-positive area increased post-TBI. However, Prussian Blue-positive areas were not clearly evident in any of the provided images.

The Prussian Blue staining was diffuse in most cases, and appears as a grey-blue discoloration of the tissues. We provide additional images of the Prussian Blue stained tissues. Additionally, we provide the iron assays of the liver tissue after both levels of radiation exposure, that provide quantification of the amount of iron increased in the tissues.
